# Umbelliferone and eriodictyol suppress the cellular entry of SARS-CoV-2

**DOI:** 10.1186/s13578-023-01070-y

**Published:** 2023-06-28

**Authors:** Fang-Ju Cheng, Chien-Yi Ho, Tzong-Shiun Li, Yeh Chen, Yi-Lun Yeh, Ya-Ling Wei, Thanh Kieu Huynh, Bo-Rong Chen, Hung-Yu Ko, Chen-Si Hsueh, Ming Tan, Yang-Chang Wu, Hui-Chi Huang, Chih-Hsin Tang, Chia-Hung Chen, Chih-Yen Tu, Wei-Chien Huang

**Affiliations:** 1 Center for Molecular Medicine, China Medical University Hospital, Taichung, 404 Taiwan; 2 Graduate Institute of Biomedical Sciences, China Medical University, Taichung, 404 Taiwan; 3 School of Medicine, China Medical University, Taichung, 404 Taiwan; 4 Department of Biomedical Imaging and Radiological Science, China Medical University, Taichung, 404 Taiwan; 5 Division of Family Medicine, Physical Examination Center, China Medical University Hsinchu Hospital, Hsinchu, 302 Taiwan; 6 Department of Medical Research, China Medical University Hsinchu Hospital, Hsinchu, 302 Taiwan; 7 Department of Plastic Surgery, and Innovation Research Center, Show Chwan Memorial Hospital, Changhua, 500 Taiwan; 8 Graduate Institute of Biomedical Engineering, National Chung Hsing University, Taichung, 402 Taiwan; 9 Department of Food Science and Biotechnology, National Chung Hsing University, Taichung, 402 Taiwan; 10 Cognitive Science, University of California San Diego, San Diego, CA 92093 USA; 11 Taichung Girls’ Senior High School, Taichung, 403 Taiwan; 12 Chinese Medicine Research and Development Center, China Medical University Hospital, Taichung, 404 Taiwan; 13 Graduate Institute of Integrated Medicine, China Medical University, Taichung, 404 Taiwan; 14 Department of Medical Laboratory Science and Biotechnology, Asia University, Taichung, 413 Taiwan; 15 Department of Chinese Pharmaceutical Science and Chinese Medicine Resources, China Medical University, Taichung, 404 Taiwan; 16 Division of Pulmonary and Critical Care Medicine, Department of Internal Medicine, China Medical University Hospital, Taichung, 404 Taiwan; 17 Department of Respiratory Therapy, China Medical University, Taichung, 404 Taiwan; 18 Drug Development Center, China Medical University, Taichung, 404 Taiwan

**Keywords:** *Artemisia argyi*, Eriodictyol, Umbelliferone, TMPRSS2, ACE2, SARS-CoV-2 variants

## Abstract

**Background:**

*Artemisia argyi (A. argyi),* also called Chinese mugwort, has been widely used to control pandemic diseases for thousands of years since ancient China due to its anti-microbial infection, anti-allergy, and anti-inflammation activities. Therefore, the potential of *A. argyi* and its constituents in reducing the infection with severe acute respiratory syndrome coronavirus 2 (SARS-CoV-2) was investigated in this study.

**Results:**

Among the phytochemicals in *A. argyi*, eriodictyol and umbelliferone were identified to target transmembrane serine protease 2 (TMPRSS2) and angiotensin-converting enzyme 2 (ACE2) proteins, the essential factors for the cellular entry of SARS-CoV-2, in both FRET-based enzymatic assays and molecular docking analyses. These two ingredients of *A. argyi* suppressed the infection of ACE2-expressed HEK-293 T cells with lentiviral-based pseudo-particles (Vpp) expressing wild-type and variants of SARS-CoV-2 spike (S) protein (SARS-CoV-2 S-Vpp) via interrupting the interaction between S protein and cellular receptor ACE2 and reducing the expressions of ACE2 and TMPRSS2. Oral administration with umbelliferone efficiently prevented the SARS-CoV-2 S-Vpp-induced inflammation in the lung tissues of BALB/c mice.

**Conclusions:**

Eriodictyol and umbelliferone, the phytochemicals of *Artemisia argyi*, potentially suppress the cellular entry of SARS-CoV-2 by preventing the protein binding activity of the S protein to ACE2.

**Supplementary Information:**

The online version contains supplementary material available at 10.1186/s13578-023-01070-y.

## Introduction

The global pandemic of coronavirus disease 2019 (COVID-19) caused by severe acute respiratory syndrome coronavirus 2 (SARS-CoV-2) has been a serious health-threatening issue in most countries [1]. The genomic evolution of SARS-CoV-2 creates diverse mutant strains, which become more transmittable and resistant to immune attack and anti-virus treatments [2]. SARS-CoV-2 variants were classified into variants of interest (VOIs) and variants of concern (VOCs) by the World Health Organization (WHO) according to their transmission rate, disease severity, therapeutic response, and healthy problem [3]. For example, the Omicron variant, identified as the lineage B.1.1.529 in South Africa in November 2021, has been viewed as one of the circulating and high lethal VOIs associated with its rapid human-to-human transmission, increased risk of reinfection, and resistance to vaccines [4–6].

SARS-CoV-2 belongs to the family of highly diverse coronaviruses (CoVs) constituted by positive-sense and single-strand RNA [7]. For cellular infection, SARS-CoV-2 utilizes various factors for the entry of host cells, translation of RNA genome, and replication cycle, ultimately releasing viral progeny. The cellular access of SARS-CoV-2 relies on its receptor engagement of spike (S) protein with the specific host receptor angiotensin-converting enzyme2 (ACE2) [8]. The receptor binding domain (RDB) of the S1 subunit of S protein directly mediates viral attachment to ACE2, while S2 is cleaved by the membrane proteases and exposed the fusion peptide (FP) for ACE2 engagement and membrane fusion [9, 10]. Transmembrane serine protease 2 (TMPRSS2) and cathepsin L are typically necessary to present the FP for membrane fusion with host cells [9, 11]. In addition, the S protein can be cleaved into S1 and S2 subunits by the proprotein convertase furin for the anchorage at the cellular membrane during the viral biosynthesis in the virus-producing cells, facilitating the virus entry [12, 13]. SARS-CoV-2 next undergoes clathrin-mediated endocytosis to gain access into cells [11]. Following virus entry, the viruses release, replicate, and transcribe their genomic RNA following cellular entry to produce essential structural proteins that compose new and complete viral particles [14, 15]. Since SARS-CoV-2 has evolved into diverse variants, the therapeutic effectiveness of anti-virus treatments is limited and scarce. It is imperative to explore the potential protective treatments or integrated approaches against COVID-19.

*Artemisia argyi* (*A. argyi*), also named Chinese mugwort, belongs to the family of *Asteraceae.* This herb has been used to control pandemic diseases by burning dried herbs for thousands of years since ancient China [16] and still is a renowned traditional Chinese medicine (TCM) and food currently used in the Far East. *A. argyi* contains a broad-spectrum profile of bioactive compounds, including flavonoids, terpenoids, and caffeoylquinic acids, and contributes to anti-microbial, anti-allergic, anti-diabetic, and anti-inflammatory activities by modulating the immune system or defending oxidative stress [17, 18]. In addition, this TCM was also shown to suppress the proliferation of tumor cells with low cytotoxicity to normal cells [19, 20]. Recently, *A. argyi* has been implied as a potential TCM against severe acute respiratory syndrome (SARS), middle east respiratory syndrome (MERS), and COVID-19 [21, 22]. Nevertheless, its activity against the infection with SARS-CoV2 has not been demonstrated yet, and the active phytochemicals and underlying mechanisms also remain unclear.

## Results

### Eriodictyol and umbelliferone, two constituents in Artemisia argyi, suppressed the enzymatic activities involved in the cellular entry of SARS-CoV-2

**Fig. 1:**
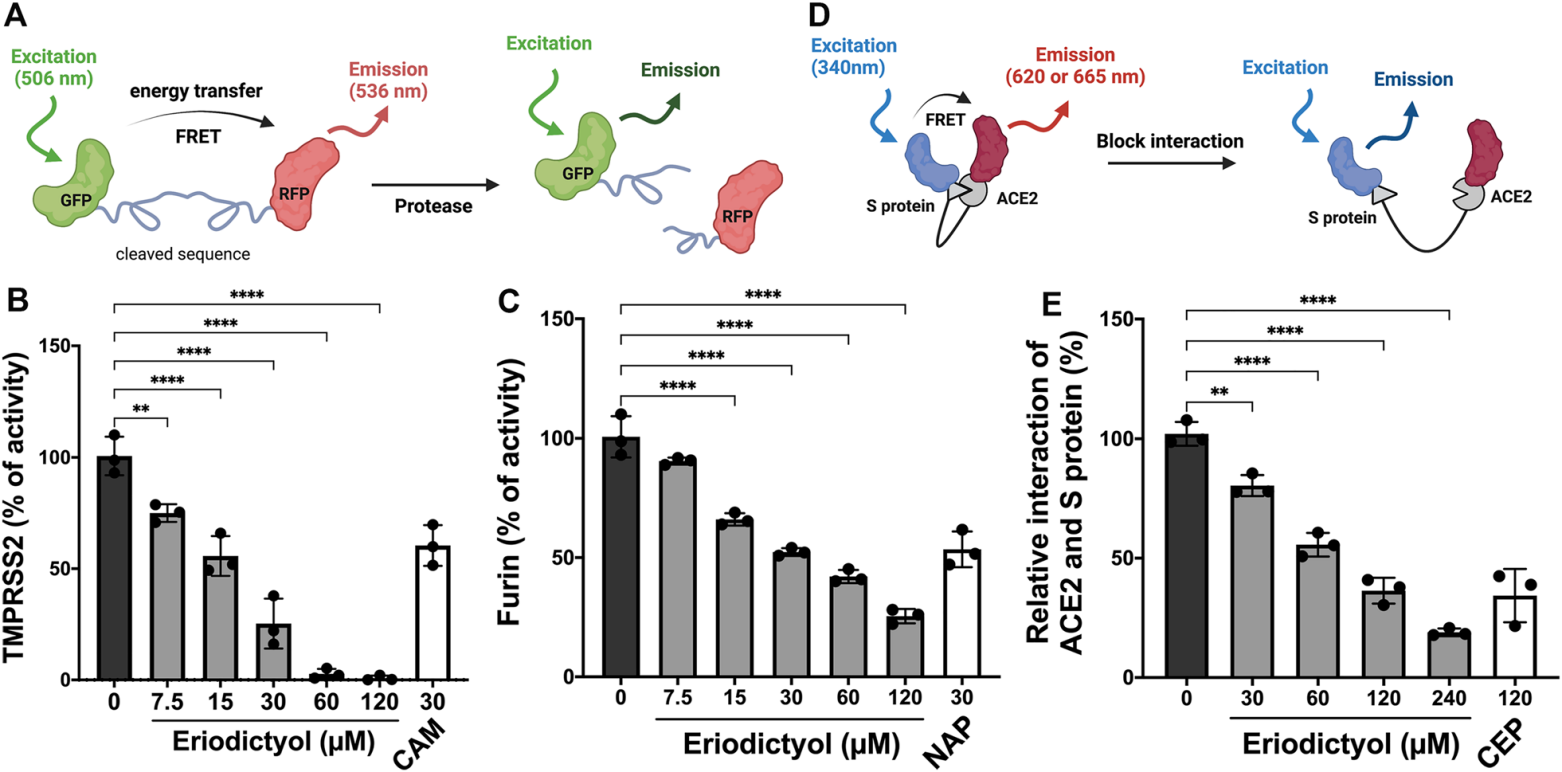
Eriodictyol suppresses the membrane-binding mechanisms of the SARS-CoV-2 spike protein. FRET-based enzymatic activity assays **A** were performed to examine the activity of TMPRSS2 **B** and furin **C** in the presence of eriodictyol at the indicated concentrations. FRET-based protein interaction assays **D** were employed to test the inhibitory effect of eriodictyol on the protein interaction between S protein and ACE2 **E**. Camostat (CAM), naphthofluorescein (NAP), and cepharanthine (CEP) were used as the positive control for the inhibitions of TMPRSS2 and furin activities and the binding between S protein and ACE2, respectively. Data are shown as mean ± SEM from three independent experiments with triplicates. ** *p* < 0.01 and **** *p* < 0.0001

To identify the bioactive compounds with the potential against the enzymatic activities for S protein priming or viral replication, 14 known ingredients of *A. argyi* [18] were subjected to fluorescence resonance energy transfer (FRET)-based enzymatic activity assays, which principle was illustrated in Fig. 1A [23]. As shown in Table 1, the enzymatic activity of TMPRSS2 was dramatically attenuated by eriodictyol (100.0% ± 0.3%), umbelliferone (74.1% ± 3.5%), and 13-Oxo-9E,11E-octadecadienoic acid (13-Oxo-ODE) (76% ± 4.7%) at 60 μM. However, none of these compounds at 60 μM suppressed more than 50% of 3CL^pro^ (Mpro) activity (Table 1), which is the main protease in cleaving coronavirus polyprotein for the replication of SARS-CoV [16]. 13-Oxo-ODE is a metabolite from de-hydrogenation of 13-hydroxyoctadecadienoic acid (13-HODE), an oxidized product of linoleic acid catalyzed by 12/15-lipoxygenase (LOX) [24, 25], and has been reported as an endogenous ligand for nuclear hormone receptor peroxisome proliferation-activated receptor gamma (PPARγ) involved in inflammatory bowel disease [26, 27]. But the clinical relevance of 13-Oxo-ODE as a potential contributor to nonalcoholic fatty liver disease [11] led us to focus on eriodictyol and umbelliferone for their anti-corona virus activity. Eriodictyol not only repressed the enzymatic activity of TMPRSS2 (Fig. 1B) but also abolished furin activity (Fig. 1C) in a dose-dependent manner. Following the priming of the S protein by TMPRSS2 or furin, the cleaved S protein interacts with the human cellular receptor ACE2 [28]. Another FRET assay, which principle was illustrated in Fig. 1D [23], further showed the inhibitory effect of eriodictyol on the interaction between S protein and ACE2 in a dose-dependent manner (Fig. 1E). Taken together, eriodictyol and umbelliferone in *A. argyi* potentially suppressed the enzymatic activities of TMPRSS2 or furin to reduce the S1/S2 priming of S protein and interfered the interaction between S protein and ACE2.

**Table 1:** Regulations of TMPRSS2 and 3CLpro by the phytochemicals in *Artemisia argyi* in FRET-based enzymatic activity assays

Compounds	Structure	Molecular formula	Suppression level of TMPRSS2 activity^a^	*p*-value^b^	Suppression level of 3CL^pro^ activity^a^	*p*-value^c^
13-Oxo-9E, 11E-octadecadienoic acid		C_18_H_30_O_3_	76.0% ± 4.7%	< 0.001	39.5% ± 4.7%	< 0.001
5,7,3'-Trihydroxy-6,4',5'-trimethoxyflavone		C_18_H_16_O_8_	12.5% ± 9.1%	0.127	6.0% ± 4.1%	0.108
beta-Rhamnocitrin		C_16_H_12_O_7_	43.8% ± 22.8%	0.053	− 4.3% ± 3.2%	0.145
Dammaradienyl acetate		C_32_H_52_O_2_	− 9.7% ± 2.0%	0.005	− 4.5% ± 10.3%	0.572
Daphnetin		C_9_H_6_O_4_	37.8% ± 10.1%	0.006	7.9% ± 0.7%	< 0.001
Daucosterol		C_35_H_60_O_6_	9.4% ± 3.7%	0.027	2.1% ± 0.3%	0.033
Eriodictyol		C_15_H_12_O_6_	100.0% ± 0.3%	< 0.001	− 1.7% ± 0.4%	0.073
Eupatilin		C_18_H_16_O_7_	-1.3% ± 0.3%	0.267	5.1% ± 5.1%	0.236
Hispidulin		C_16_H_12_O_6_	13.0% ± 0.7%	< 0.001	-2.5% ± 0.1%	0.018
Jaceosidin		C_17_H_14_O_7_	51.8% ± 5.2%	< 0.001	11.6% ± 3.6%	0.012
Linarin		C_28_H_32_O_14_	-6.6% ± 1.2%	0.007	1.6% ± 0.4%	0.076
Octacosanoic acid		C_28_H_56_O_2_	− 43.3% ± 40.2%	0.203	− 1.0% ± 4.2%	0.775
Stigmasterol		C_29_H_48_O	8.4% ± 7.6%	0.197	3.6% ± 3.8%	0.255
Umbelliferone		C_9_H_6_O_3_	74.1% ± 3.5%	< 0.001	− 4.0% ± 0.9%	0.012

^a^The relative suppression compared to control

^b^Statistics difference for the suppression of TMPRSS2 activity

^c^Statistics difference for the suppression of 3CL^pro^ activity

### Molecular docking analysis of eriodictyol and umbelliferone with SARS-CoV-2 proteins

**Fig. 2:**
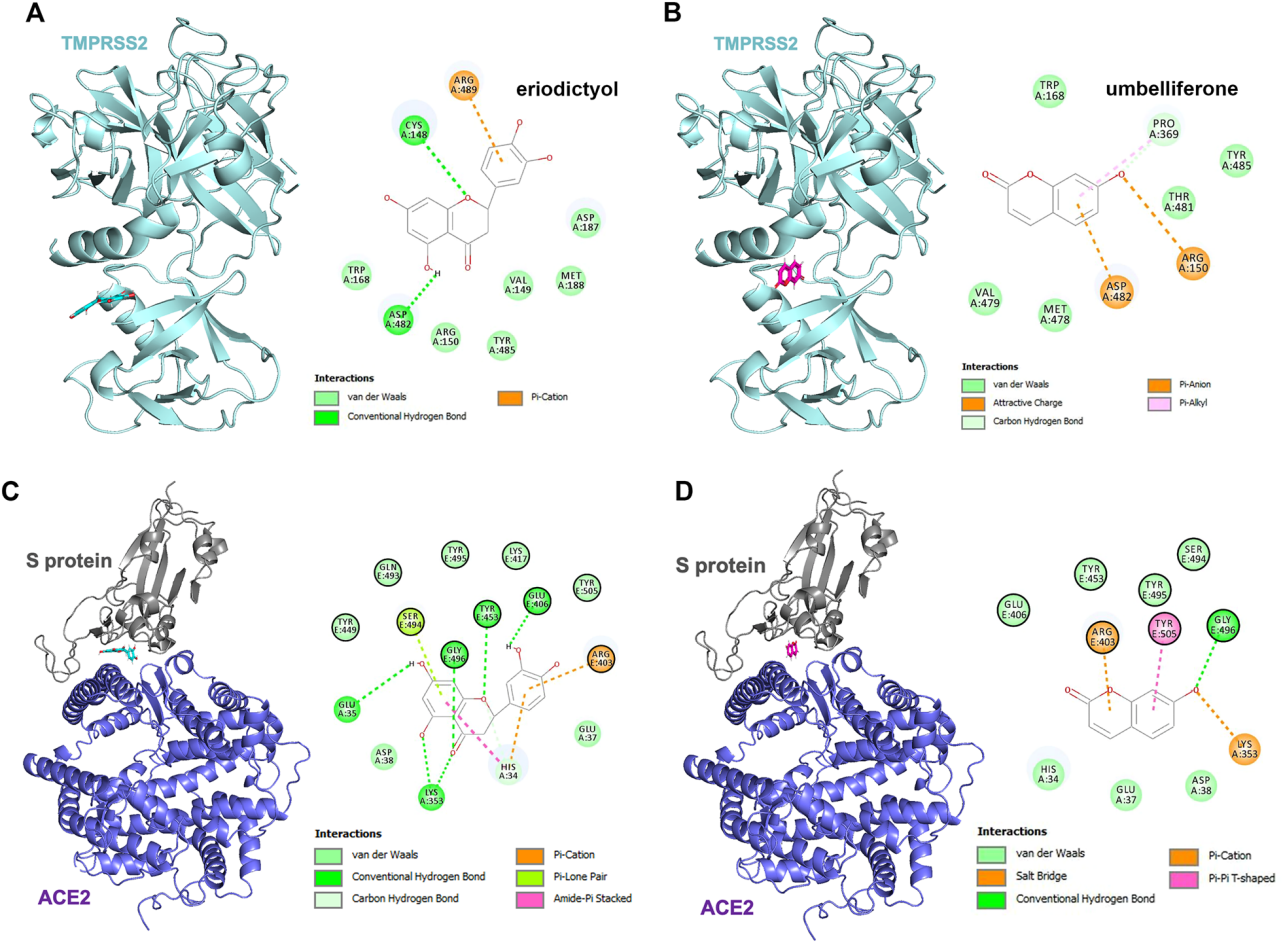
The interactions of eriodictyol and umbelliferone with TMPRSS2, ACE2, and SARS-CoV-2 S proteins. The predicted binding affinities of TMPRSS2 (PDB ID: 7MEQ) and the interface between ACE2 and S protein (PDB ID: 6M0J) with eriodictyol **A** and** C** and umbelliferone **B** and** D** were investigated by using BIOVIA Discovery Studio. The rounds with black rings (right of **C** and **D** indicated the amino acid residues of S protein interacting with eriodictyol or umbelliferone

Since eriodictyol and umbelliferone suppressed the enzymatic activity of TMPRSS2 and S protein/ACE2 interaction, the binding modes of these two compounds in the catalytic pockets of TMPRSS2 and the interface between the S protein and ACE2 were performed with Discovery Studio. The results indicated that eriodictyol (Fig. 2A) and umbelliferone (Fig. 2B) showed binding affinity to the catalytic pockets of TMPRSS2, and the changes in energy value were − 13.05 kcal/mol (eriodictyol/TMPRSS2) and − 14.82 kcal/mol (umbelliferone/TMPRSS2), respectively (Table 2). Furthermore, the interface between the S protein and human receptor ACE2 was also disrupted by these two compounds (Figs. 2C, D) with the free energy decreases of − 33.478 and -36.539 kcal/mol for eriodictyol/S protein-ACE2 and umbelliferone/S protein-ACE2 complexes, respectively (Table 2). Based on these results, eriodictyol and umbelliferone could be the phytochemicals in *A. argy* capable of targeting the potential residues (Table 2) of multiple proteins associated with the cellular entry of SARS-CoV-2.

**Table 2:** The binding energy and interaction sites of TMPRSS2, ACE2, and SARS-CoV-2 S proteins with eriodictyol and umbelliferone

Compounds	Proteins	Energy value (kcal/mol)	Amino acids	Interactions
Eriodictyol	TMPRSS2	− 13.05	Cys 148	Hydrogen bond (2.60 Å)
			Asp 482	Hydrogen bond (2.09 Å)
			Arg 489	Pi-Cation (3.73 Å)
	S protein-ACE2	− 33.478	Arg 403^a^	Pi-Cation (2.89 Å)
			Glu 406^a^	Hydrogen bond (2.03 Å)
			Tyr 453^a^	Hydrogen bond (2.24 Å)
			Ser 494^a^	Pi-Lone pair (2.69 Å)
			Gly 496^a^	Hydrogen bond (2.02 Å)
			His 34^b^	Pi-Cation (3.47 Å); Hydrogen bond (2.55 Å); Amide-Pi Stacked (4.48 Å)
			Glu 35^b^	Hydrogen bond (2.03 Å)
			Lys 353^b^	Hydrogen bond (1.83 Å, 1.93 Å)
Umbelliferone	TMPRSS2	− 14.82	Arg 150	Attractive charge (4.93 Å)
			Pro 369	Hydrogen bond (2.41 Å); Pi-Alkyl (4.54 Å)
			Asp 482	Pi-Anion (3.99 Å)
	S protein-ACE2	− 36.539	Arg 403^a^	Pi-Cation (4.03 Å)
			Gly 496^a^	Hydrogen bond (2.61 Å)
			Tyr 505^a^	Pi-Pi T shaped (5.97 Å)
			Lys 353^b^	Salt bridge (1.65 Å)

^a^Interaction with S protein

^b^Interaction with ACE2

### A. argyi and its active compounds downregulate ACE2 and TMPRSS2 expressions in lung epithelial cells

**Fig. 3:**
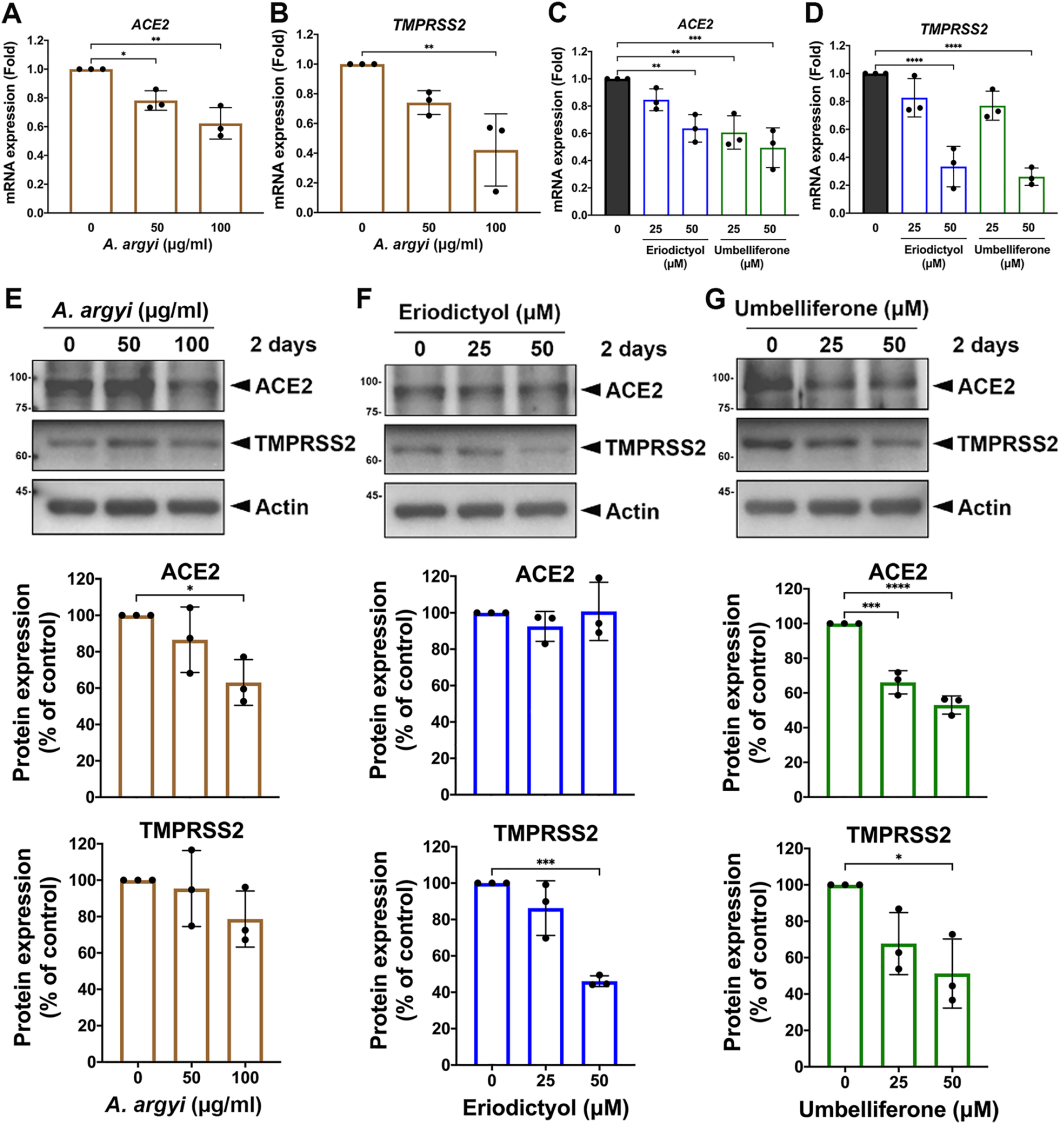
Eriodictyol and umbelliferone inhibited the expressions of ACE2 and TMPRSS2 in lung epithelial cells. Beas 2B cell lines were treated with *A. argyi*, eriodictyol, and umbelliferone at the indicated concentrations for 2 days. The cell lysates and total RNA harvested from Beas 2B cells were subjected to RT-qPCR **A-D** and Western blot analysis **E–G** analyses to examine the effects of tested compounds on the mRNA and protein levels of ACE2 and TMPRSS2, respectively. The quantitated data of protein level was shown below. Data were shown as mean ± SEM from three independent experiments with triplicates. * *p* < 0.05, ** *p* < 0.01, *** *p* < 0.001, and **** *p* < 0.0001

The cytotoxic activities of *A. argyi*, eriodictyol, and umbelliferone were next examined in Beas 2B lung epithelial cells using MTT assays. Cytotoxicity concentration 50% (CC_50_) values of *A. argyi*, eriodictyol, and umbelliferone were 4471 μg/ml (Additional file 1: Fig. S1A), 212.6 μM (Additional file 1: Fig. S1B), and 302.4 μM (Additional file 1: Fig. S1C), respectively. Based on the level of CC_50_, we further addressed whether the expressions of ACE2 and TMPRSS2 are also affected by these potential agents in preventing the cellular entry of SARS-CoV-2. *A. Argyi*, eriodictyol, and umbelliferone significantly suppressed the and mRNA expressions of ACE2 and TMPRSS2 (Fig. 3A–D). While *A. argyi* suppressed ACE2 protein expression at 100 μg/ml (Fig. 3E) and eriodictyol reduced TMPRSS2 protein level at 50 μM (Fig. 3F), umbelliferone showed more potent inhibitory effects on both ACE2 and TMPRSS2 protein expressions in a dose-dependent manner (Fig. 3G). These results suggest that *A. argyi,* eriodictyol, and umbelliferone can inhibit SARS-CoV-2 infection by mainly repressing the expressions of cellular ACE2 and TMPRSS2 with different specificity and potency.

### *A. argyi,* eriodictyol, and umbelliferone broadly suppress the cell entry of SARS-CoV-2 S-Vpp variants

**Fig. 4:**
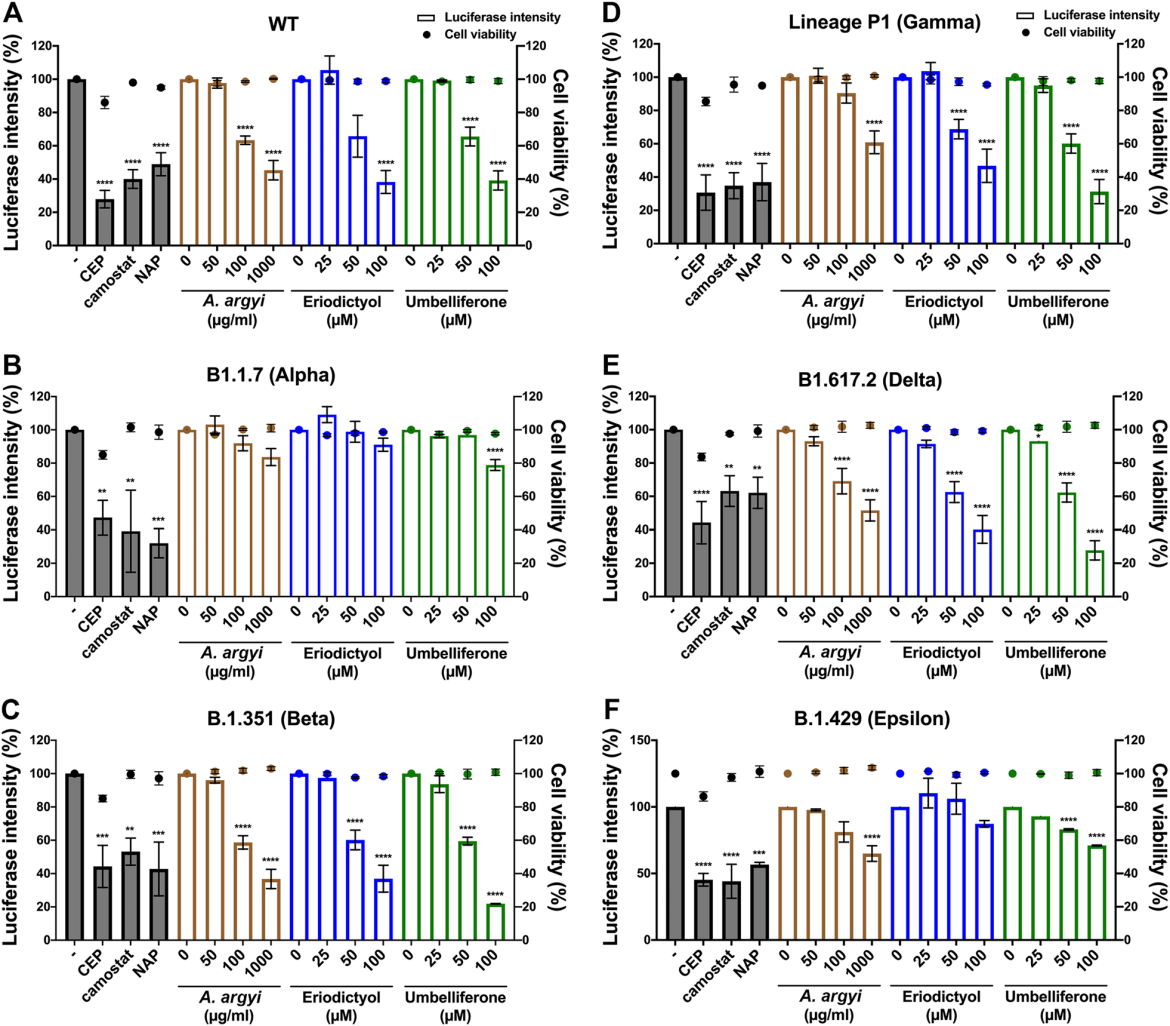
The inhibitory effects of *A. argyi*, eriodictyol, and umbelliferone on the cellular entry of Vpp of SARS-CoV-2 S variants. HEK-293 T cells expressing ACE2 were infected with SARS-CoV-2 S protein pseudo viral variants with luciferase, including wild type **A** B1.1.7 (alpha) **B** B.1.351 (Beta) **C** Lineage P1 (Gamma) **D** B1.617.2 (Delta) **E** and B.1.429 (Epsilon) **F** after treatments with *A. argyi*, eriodictyol or umbelliferone at the indicated concentrations for 2 days, and the luciferase activities were measured to determine the infection rate with the pseudoviruses. Additionally, the cell viability also was examined in CCK-8 assays. Camostat (CAM), naphthofluorescein (NAP), and cepharanthine (CEP) were used as the positive controls for the inhibitions of TMPRSS2 and furin activities and the binding between S protein and ACE2, respectively. Data were shown as mean ± SEM from three independent experiments with triplicates. ** *p* < 0.01, *** *p* < 0.001, and **** *p* < 0.0001

The above data suggest that eriodictyol and umbelliferone could prevent the cellular entry of SARS-CoV-2 through multiple mechanisms, including inhibitions of cellular TMPRSS2 activity, cellular ACE2 and TMPRSS2 expressions, and S protein/ACE2 interaction. Therefore, we further assessed the efficacy of *A. argyi* extracts and its phytochemicals eriodictyol and umbelliferone in blocking the cellular entry of SARS-CoV-2 S protein-pseudo-type lentiviral particles (SARS-CoV-2 S-Vpp). HEK-293 T cells stably expressing ACE2 protein were employed to test the cellular entry of SARS-CoV-2 Vpp with S protein variants following the pretreatments with tested drugs. The results displayed that the infections with most SARS-CoV-2 S-Vpp variants, including wild type (WT) (Fig. 4A), B.1.351 (Beta) (Fig. 4C), Lineage P1 (Gamma) (Fig. 4D), and B1.617.2 (Delta) (Fig. 4E) were dramatically repressed by the treatments with *A. argyi*, eriodictyol, and umbelliferone in a dose-dependent manner without affecting the infection with VSVG control (Additional file 2: Fig. S2) and the cell viability. However, the infections with B1.1.7 (Alpha) (Fig. 4B) and B.1.429 (Epsilon) (Fig. 4F) were not affected by *A. argyi* extracts. The inhibitory concentration 50% (IC_50_) values of *A. argyi*, eriodictyol, and umbelliferone in antiviral activity are shown in Table 3. The IC_50_ values of *A. argyi*, eriodictyol, and umbelliferone in the detected viruses, except B1.1.7 (Alpha) and B.1.429 (Epsilon), were around 332 ~ 1534 μg/ml, 88 ~ 126 μM, and 67 ~ 100 μM, respectively. These findings suggested that *A. argyi* can inhibit infections with SARS-CoV-2.

**Table 3:**
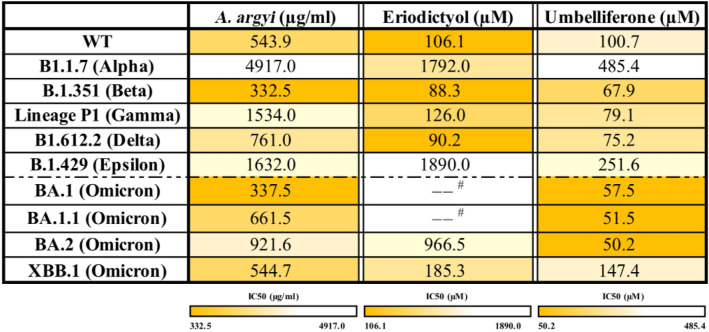
IC_50_ value of *A. argyi*, eriodictyol, and umbelliferone against the Vpp of SARS-CoV-2 variants

^#^Undetectable

**Fig. 5:**
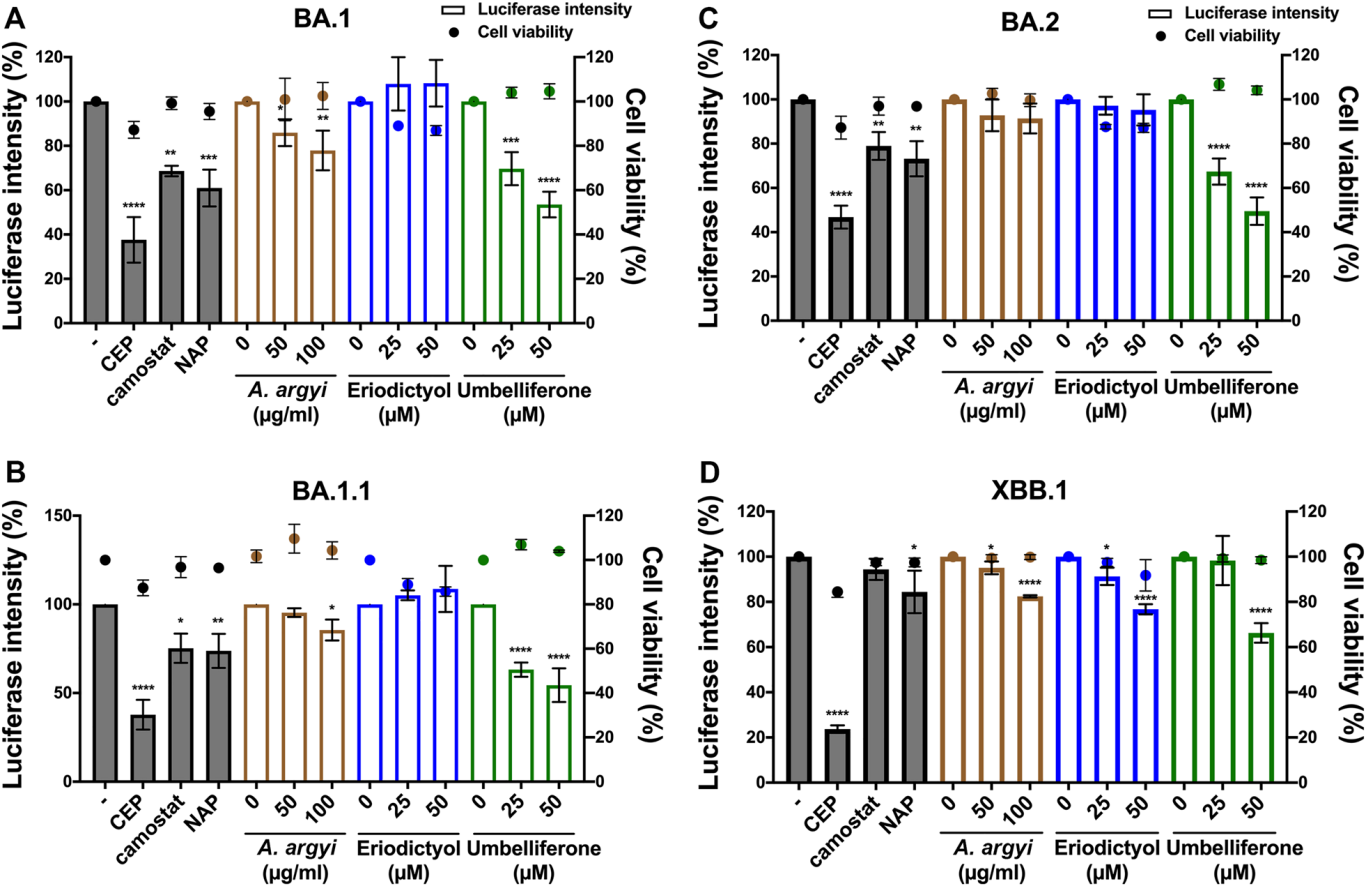
The inhibition effects of *A. argyi*, eriodictyol, and umbelliferone on the cellular entry of SARS-CoV-2 Omicron variants. HEK-293 T cells expressing ACE2 were infected with SARS-CoV-2 Omicron pseudo viral variants with luciferase, including BA.1 **A** BA.1.1 **B** BA.2 **C** and XBB.1 **D** after treatments with *A. argyi*, eriodictyol, or umbelliferone at the indicated concentrations for 2 days, and the luciferase activities were measured to determine the infection rate with these pseudoviruses. Additionally, the cell viability also was examined in CCK-8 assays. Camostat (CAM), naphthofluorescein (NAP), and cepharanthine (CEP) were used as the positive controls for the inhibitions of TMPRSS2 and furin activities and the binding between S protein and ACE2, respectively. Data are shown as mean ± SEM from three independent experiments with triplicates. * *p* < 0.05, ** *p* < 0.01, *** *p* < 0.001, and **** *p* < 0.0001

Omicron (B.1.1.529), first reported in November 2021, is a highly mutated variant of SARS-CoV-2 and has become the predominant viral variant of concern (VOC) worldwide. The inhibitory effects of tested compounds on the cellular entry of SARS-CoV-2 S-Vpp of Omicron variants were next examined in HEK-293 T cells expressing ACE2 proteins. The results showed that umbelliferone significantly and dose-dependently suppressed the cellular entry of all tested Omicron variants BA.1 (Fig. 5A), BA.1.1 (Fig. 5B), BA.2 (Fig. 5C), and XBB.1 (Fig. 5D). *A. argyi* also significantly reduced the infections with BA.1, BA1.1, and XBB.1 variants. However, eriodictyol only slightly interfered with the infection with XBB.1 but not other Omicron variants. Our findings suggested that umbelliferone can be a potential agent more selective against the variants of SARS-CoV-2 Omicron with IC_50_ values of around 50 ~ 147 μM (Table 3).

### Umbelliferone effectively suppressed the lung inflammation of mice induced by SARS-CoV-2 S-Vpp of WT or Omicron variants

**Fig. 6:**
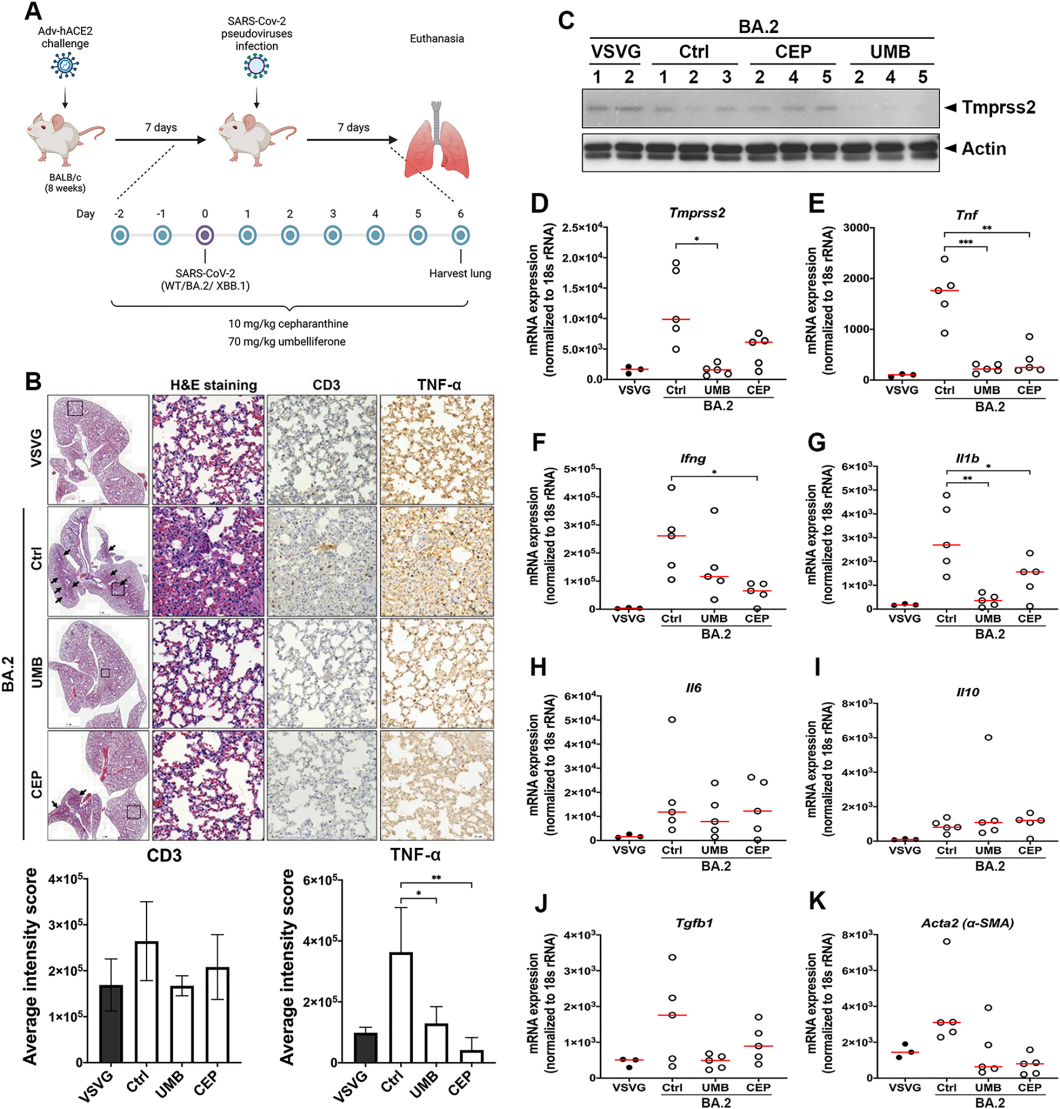
Umbelliferone represses the Omicron BA.2-associated pulmonary inflammation in vivo. **A** Schematics of the administration process of the mouse model. The mouse model was created by bioRENDER. The mice infected with VSVG or Omicron BA.2 pseudoviruses were sacrificed after oral administration with sterilized water (Ctrl), 70 mg/kg umbelliferone (UMB), and 10 mg/kg cepharanthine (CEP) for 9 days, and lungs were subsequently harvested for the examinations of histopathological changes in H&E staining **B**, lymphocyte infiltration and TNF-α protein expression in IHC staining **B**, TMPRSS2 protein in Western blot analysis **C**, and the mRNA levels of indicated genes in RT-qPCR **D-K**, respectively. * *p* < 0.05, ** *p* < 0.01, and *** *p* < 0.001

Since umbelliferone showed promising activity against the cellular entry of SARS-CoV-2 S-Vpp in vitro, its inhibitory effects on SARS-CoV-2-associated inflammation in lung tissues were addressed in a mice model. As illustrated in Fig. 6A, BALB/c mice expressing human ACE2 were pre-treated with umbelliferone 70 mg/kg for 2 days, followed by challenge with WT, BA.2, or XBB.1 variants of SARS-CoV-2 S-Vpp and continuous treatment with umbelliferone for another 7 days. Compared to the infection of SARS-CoV-2 S-Vpp WT (Additional file 3: Fig. S3A), infections with BA.2 (Fig. 6B) and XBB.1 (Additional file 4: Fig. S4A) variants more obviously elicited inflammation as evidenced by the formation of granuloma (indicated with the arrow) in H&E staining and the inductions of inflammatory gene expressions, including *Tnf*, *Infg*, *Il1b*, *Il6*, *Il10*, and *Tgfb1* in RT-qPCR analysis (Additional file 5: Figs. S5A–F). IHC staining with anti-CD3 and TNF-α antibodies further supported the SARS-CoV-2 S-Vpp-induced infiltration of immune cells and inflammation (Fig. 6B and Additional files 3, 4: Figs. S3A and S4A). The increases in the mRNA level of alpha-smooth muscle actin (α-SMA), a marker for fibrogenesis [29], further revealed the tendency of lung tissues to form lung fibrosis by the infection with the pseudo viral particles (Additional file 5: Fig. S5G). Similar to the effect in vitro (Fig. 3), pretreatment with umbelliferone suppressed the protein and mRNA expression of TMPRSS2 in lung tissues (Figs. 6C, D, and Additional files 3, 4: Figs. S3B, C and S4B, C). Also, it reduced the granuloma formation, CD3-positive immune cell infiltration, and TNF-α expression (Fig. 6B and Additional file 3: Figs. S3A and Additional file 4: S4A). The decreases in the mRNA expressions of inflammatory genes (Fig. 6E–J and Additional files 3, 4: Figs. S3D–I and S4D–I) and α-SMA expressions (Fig. 6K and Additional files 3, 4: Figs. S3J and S4J) further supported the potential of umbelliferone in preventing infection with SARS-CoV-2 variants in vivo.

### Eriodictyol decreases the replication of SARS-CoV-2 by enzymatic inhibition of RdRp

After the entry and uncoating of SARS-CoV-2, 3-chymotrypsin-like protease (3CL^pro^) and papain-like protease (PL^pro^) are the virus-encoded cysteine proteases for cleaving the viral polyprotein and then generating nonstructural proteins during viral replication [30, 31]. RNA-dependent RNA polymerase (RdRp; also known as nsp12) is a viral replicase for synthesizing complementary RNA during the transcriptional cycle of SARS-CoV-2 [32]. Therefore, we further examined the inhibitory effects of tested compounds on the enzymatic activity of 3CL^pro^, PL^pro^, and, RdRp by performing FRET-base enzymatic activity assays and found that eriodictyol dramatically reduced the activities of these three viral enzymes (Additional file 6: Figs. S6A–C). However, the 50% inhibition concentrations of eriodictyol for 3CL^pro^ and PL^pro^ were over 240 μM. These results displayed that *A. argyi* and its phytochemicals may interfere with the cellular entry and RNA replication of SARS-CoV-2 at relatively lower doses and showed inhibitory effects on the viral protease activities at higher doses.

## Discussion

**Fig. 7:**
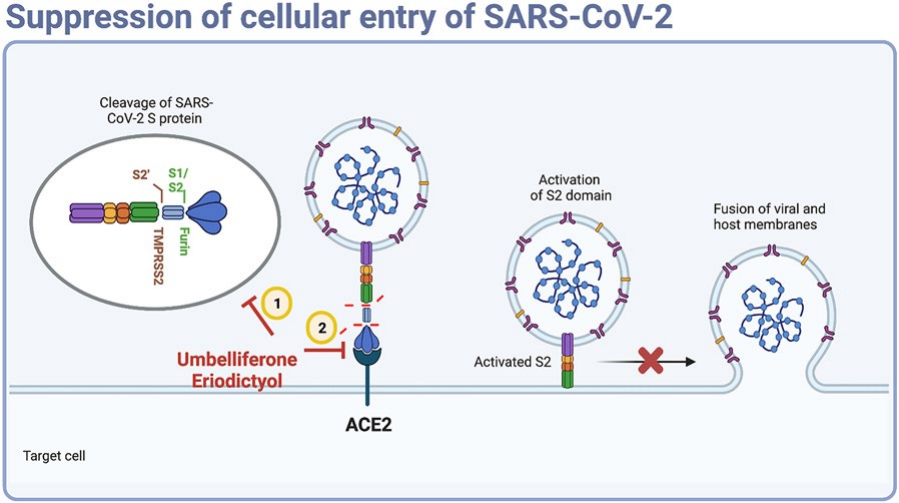
Schematics of the working model of umbelliferone and eriodictyol in inhibiting SARS-CoV-2 infection. Umbelliferone and eriodictyol attenuate the enzymatic activities of furin and TMPRSS2, which cleaves and primes SARS-CoV-2 spike protein, suppressing the cellular entry of SARS-CoV-2 variants. The photograph was created by bioRENDER

Until the end of 2022, the COVID-19 pandemic has caused more than 60 million confirmed cases and 6 million deaths worldwide. The emergency of SARS-CoV-2 variants, which not only facilitate viral replication, transmission, and immune escape but also weaken the protective ability of recently developed vaccines [2, 33], generates more concerns about the prevention of SARS-CoV-2 infection. TCMs have been explored as potential therapeutics against COVID-19 and can improve the efficacy of standard treatments while diminishing disease deterioration [34, 35]. For example, Taiwan Chingguan Yihau (NRICM101) has been shown to disrupt virus invasion and host inflammation in patients with SARS-CoV2 infections [36, 37]. Our data showed that treatment with *A. argyi*, a well-known herbal medicine used in the Far East, can be a new strategy to prevent infections with multiple variants of SARS-CoV2 by suppressing their cellular entry as well as viral replication via targeting cellular proteins TMPRSS2 and ACE2 and viral protein RdRp, respectively (Fig. 7 and Additional file 6: Fig. S6D).

The binding affinity of jaceosidin and eupatilin, two phytochemicals in *A. argyi*, to SARS-CoV-2 3CL^pro^ has been predicted in molecular docking simulation [38]. In the present study, however, several flavonoids, including 5,7,3'-Trihydroxy-6,4',5'-trimethoxyflavone, β-Rhamnocitrin, eriodictyol, eupatilin, hispidulin, and jaceosidin containing the basic skeleton of bezno-γ-pyrene [39] did not show the inhibitory effect on the activity of SARS-CoV-2 3CL^pro^ (Table 1), suggesting that the flavonoids in *A. argyi* mainly target the cellular entry of SARS-CoV-2 to decrease the deterioration from COVID-19.

Although the cellular entry of SARS-CoV-2. B1.1.7 (Alpha) also depends on the binding to human receptor ACE2, the transmissibility of B1.1.7 is lower than that of other classified SARS-CoV-2 mutants [40]. The S1-receptor binding domain (RBD) of B1.1.7 may maintain an unfavorable conformation before ACE2 engagement, causing its weak binding affinity to human ACE2 [41, 42]. It explains the fewer effects of *A. argyi*, eriodictyol, and umbelliferone on the cellular entry of SARS-CoV-2. B1.1.7 (Alpha) (Fig. 4B). Similar to our findings, many other S protein-targeting agents, such as *Echinacea purpurea* [43] and Montelukast [44], showed efficacy in preventing infections with different SARS-CoV-2 mutants except B1.1.7.

SARS-CoV-2 lineage B.1.1.529, named Omicron strain, was documented as a VOC on 26th November 2021 by WHO and became the significant viral strain in the COVID-19 pandemic. Unlike other SARS-CoV-2 variants, Omicron is marked by many mutations across the entire genome, including its spike glycoprotein gene [45], contributing to immune escape, an attenuating ability of vaccines, and a higher frequency of reinfections [5, 6]. Unlike the alpha strain, these high mutations of the Omicron S protein are associated with its increased binding efficacy to host receptor ACE2 [46]. Our data also showed that infections with Omicron variants also caused a higher frequency of inflammation in lung tissues (Fig. 6 and Additional files 3, 4, 5: Figs. S3, S4, S5). While other SARS-CoV-2 variants require the S protein priming by the host transmembrane protein TMPRSS2 for cellular entry, the infection of Omicron variants can be accomplished by an endocytic route in a TMPRSS2-independent manner [47, 48]. Our results found that both *A. argyi* and umbelliferone diminished the protein level of ACE2 (Fig. 4), accounting for their activity in blocking the cellular entry of Omicron variants (Figs. 5, 6 and Additional file 4: Fig. S4). Therefore, umbelliferone would be the critical phytochemical in *A. argyi* capable of reducing the infection with Omicron variants.

## Conclusions

In general, this study explored the potent antiviral activity of *A. argyi* and its phytochemicals (eriodictyol and umbelliferone) against multiple SARS-CoV-2 variants with the IC_50_ values ranging from 332 to 1534 µg/ml and 67 to 126 µM, respectively. Mechanistically, *A. argyi*, eriodictyol, and umbelliferone repress the enzymatic activity of TMPRSS2 and furin for priming S protein, impeding the interaction between S protein and ACE2, and inhibit the RdRp-mediated viral replication (Fig. 7 and Additional file 6: Fig. S6D). In addition, *A. argyi* and umbelliferone also showed specific activity against Omicron variants due to their inhibitory effect on ACE2 protein expression.

## Methods

### Cell lines culture

Beas 2B epithelial cells, which were established from normal human bronchus, were grown in Dulbecco’s Modified Eagle Medium (DMEM) with low glucose (1 g/L) and sodium pyruvate (Gibco), and ACE2-expressing HEK-293 T cells were cultured in DMEM/Nutrient Mixture (F-12) medium (Gibco). The culture mediums for these two cell lines contained 10% fetal bovine serum (FBS; Gibco) and 1% penicillin–streptomycin (P/S; Thermo Fisher Scientific), and both cell lines were incubated at 37 ℃ in a humidified 5% CO_2_/95% air incubator.

### Preparation of A. argyi extraction and pure compounds

The powder of *A. argyi* obtained from dry plants was dissolved and heated in sterilized H_2_O at 37 ℃ for 30 min. Eriodictyol (Cayman), umbelliferone (Sigma-Aldrich), and other ingredients of *A. argyi* listed in Table 1 were dissolved in DMSO.

### Measurement of cell viability

The cell viability was measured in MTT (Sigma-Aldrich) and cell counting kit-8 (CCK-8; Dojindo) according to the manufacturer’s protocols. Beas 2B cells (5000 cells/well) in a 96-well plate were treated with *A. argyi* solution, eriodictyol, or umbelliferone in a dose-dependent manner for 2 days and then subjected to MTT assays. ACE2-expressing HEK-293 T cells were treated with *A. argyi* solution, eriodictyol, or umbelliferone for 2 days followed by infections with the variants of SARS-CoV-2 pseudoviruses, and their viabilities were then detected in CCK-8 assays.

### Infection with pseudo-typed lentiviral particles (Vpp) of SARS-CoV-2 S protein mutants

The virus particle pseudo-typed (Vpp) of SARS-CoV-2 S protein mutants and the vesicular stomatitis-G (VSV-G) control bearing luciferase were obtained from RNA Technology Platform and Gene Manipulation Core, Academia Sinica in Taiwan. ACE2-expressing HEK-293 T cells were pre-treated with *A. argyi*, eriodictyol, and umbelliferone at the indicated concentrations for 2 days and then were infected with the pseudo lentivirus of VSVG and SARS-CoV-2 variants for 1 day. After determining the cell viability in cell counting kit-8 assay (CCK-8; Dojindo), the luciferase intensity of the lysates prepared with One-Glo^™^ Luciferase assay buffer (Promega) was measured in Luminescence Plate Reader.

### Molecular docking simulation

The structures of proteins and compounds in this study were retrieved from Protein Data Bank (PDB, https://www.rcsb.org/) and were applied to molecular docking calculation (BIOVIA Discovery Studio) to simulate the binding efficacy of tested compounds to S protein and receptor ACE2 as well as TMPRSS2.

### Fluorescence resonance energy transfer (FRET)-based enzymatic activity assay

In the examination of TMPRSS2 and Furin protease activities in the cleavage of SARS-CoV-2 spike protein, the recombinant proteins (15 μg/ml) of TMPRSS2 and Furin were pre-incubated in the assay buffer (25 mM Tris 8.0, 150 mM NaCl) with/without eriodictyol at room temperature for 30 min. Additionally, Camostat (CAM) [49] and naphthofluorescein (NAP) [50] were employed as the positive control against the activity of TMPRSS2 and furin, respectively. After adding 20 μM of the fluorescent protein substrates, the reaction of substrate cleavage was monitored continuously for 6 h by detecting mNeonGreen fluorescence (excitation: 506 nm/emission: 536 nm) using Synergy^™^ H1 hybrid multi-mode microplate reader (BioTek Instruments, Inc.) [23, 51]. The first 1 h of the reaction was used to calculate the initial velocity (V_0_). The initial velocity with each compound was calculated and normalized to DMSO control (as illustrated in Fig. 1A).

In the analysis of the enzymatic activities associated with SARS-CoV-2 replication, including 3C-like protease (3CL^pro^), papain-like protease (PL^pro^), and RNA-dependent RNA polymerase (RdRp), the tested compounds were first incubated with the recombinant proteins of these proteases at room temperature. Additionally, GC-376 (GC) [49], GRL-0617 (GRL) [52], and GS-443902 (GS, an active metabolite from remdesivir) [53], and cepharanthine [54], were employed as positive controls against the activity of 3CL^pro^, PL^pro^, RdRp, and S/ACE2 interaction, respectively. After 30 min of incubation, the fluorescent protein substrate was added to the reaction. The fluorescent signal was recorded by detecting the excitation wavelength of 505 nm and emission wavelength of 536 nm [55].

### Time-resolved fluorescence resonance energy transfer (TR-FRET) assay

The interruption effects of tested compounds on the interaction between SARS-CoV-2 Spike S1 and human ACE2 proteins were measured by using TR-FRET assays according to the manufacturer’s protocol (BPS Bioscience, Inc.) [23, 51]. Briefly, the recombinant proteins of ACE2 and SARS-CoV-2 Spike S1 were incubated with or without the tested compounds at the indicated concentrations at room temperature for 1 h. TR-FRET signals were recorded by detecting the emission at a wavelength of 620 or 665 nm with the excitation at a wavelength of 340 nm (Fig. 1D).

### In vivo infection

The 8-week-old female BALB/c mice (5 for each group) were purchased from LASCO. For pseudovirus infection, mice were first treated with 2.5 × 10^8^ PFU of Adv-hACE2 via intranasal administration. After Adv-hACE2 infections for 7 days, mice were intranasally delivered 10^5^ PFU of VSVG or SARS-CoV-2 pseudo-lentiviruses of wild-type spike protein, BA.2, and XBB.1 for 7 days [56]. Before 2 days of infections with SARS-CoV-2 pseudo-lentiviruses, mice were orally fed with water, 70 mg/kg umbelliferone, or 10 mg/kg cepharanthine for 9 days (as illustrated in Fig. 5A). At the indicated harvest time point, mice were euthanized by CO_2_ inhalation, and lungs were collected for analyzing histopathology, TMPRSS2 expression, and the mRNA levels of inflammation cytokines in immunohistochemistry, western blotting, and RT-qPCR analyses, respectively. All the procedures of the animal experiments in this study were approved by the Institutional Animal Care and Use Committee of China Medical University (CMUIACUC-2021-87) in accordance with NIH guidelines.

### Western blot analysis

Protein lysates from mouse lungs or Beas 2B lung cells prepared in RIPA buffer with protease and phosphatase inhibitors were separated in SDS-PAGE and then transferred to PVDF membranes. The membranes were blocked in 5% milk in TBST buffer (TBS with 0.1% Tween 20) and were incubated with primary antibodies against TMPRSS2 (ProteinTech), ACE2 (Genetex), or β-actin (Sigma-Aldrich) at 4 ℃ for overnight followed by the incubation with HRP-conjugated second antibody at room temperature for 1 h. After washing with TBST buffer, the immunoreactive signals were visualized using enhanced chemiluminescence with enhanced chemiluminescence (ECL) reagent.

### Quantitative reverse transcription PCR (RT-qPCR)

Total RNA from mouse lungs or Beas 2B lung cells was extracted with TRIzol reagent (Invitrogen). The first-strand cDNA synthesis was started in a 20 μl mixture with 500 ng of mRNA, dNTP, Oligo-dT, and distilled DEPC H_2_O at 65 ℃ for 5 min, and was added with 5X First-Strand Buffer, 0.5 M DTT, 200 U/μl M-MLV reverse transcriptase (Invitrogen) for the incubation at 37 ℃, 25 ℃, and 37 ℃ for 2, 10, 50 min, respectively, followed by another incubation at 70 ℃ for 15 min. The cDNA products were subjected to real-time PCR analyses with SYBR Green Master Mix to detect the gene expressions of ACE2, TMPRSS2, inflammatory cytokines, and α-SMA with specific primer sets (Additional file 7: Table S1).

### Immunohistochemistry (IHC) staining

The sections of paraffin-embedded lung tissue (5 μm) were dewaxed by xylene and rehydrated by 100%, 95%, 80%, and 75% of ethanol. These sections were incubated overnight with indicated primary antibodies and then were washed to remove unbound primary antibodies. These sections are then stained with a polymer HRP-conjugated secondary rabbit antibody for 30 min followed by a reaction with diaminobenzidine for 2 min. TissueFAXS was used to capture the images and perform the quantitative analysis.

### Statistical analysis

Data are shown as the mean ± standard error of the mean (SEM). A two-tailed t-test was used for comparisons. A p-value < 0.05 was considered statistically significant.

## Supplementary Information

**Additional file 1: Fig. S1 **The cytotoxicity of *A. argyi*, eriodictyol, and umbelliferone is illustrated in lung epithelial cells. Beas 2B cells were treated with *A. argyi ***A** eriodictyol **B**, and umbelliferone **C** at the indicated concentrations for 2 days. Then the cell viability was detected using MTT assays. The cytotoxicity concentration 50%value was calculated and displayed. Data was shown as mean±SEM from three independent experiments with triplicates.

**Additional file 2: Fig. S2 ***A. argyi*, eriodictyol, and umbelliferone did not affect the cellular entry of VSVG-pseudotyped lentivirus.HEK-293T cells expressing ACE2 were infected with VSVG-pseudotyped lentivirus encoding luciferase after treatments with *A. argyi*, eriodictyol, or umbelliferone at the indicated concentrations for 2 days, and the luciferase activities were subsequently measured to determine the infection rate with the pseudoviruses. Additionally, the cell viability also was examined in CCK-8 assays. Camostat, naphthofluorescein, and cepharanthinewere used as the positive controls for the inhibitions of TMPRSS2 and furin activities and the binding between S protein and ACE2, respectively. Data were shown as mean±SEM from three independent experiments with triplicates. *** *p*<0.001.

**Additional file 3: Fig. S3 **Umbelliferone represses the wild-type SARS-CoV-2-associated pulmonary inflammation *in vivo*.The mice infected with VSVG or wild-type SARS-CoV-2 pseudoviruses were sacrificed after oral administration with sterilized water, 70 mg/kg umbelliferone, and 10 mg/kg cepharanthinefor 9 days, and lungs were subsequently harvested for the examinations of histopathological changes in H&E staining **A** lymphocyte infiltration and TNF-α protein expression in IHC staining **A** TMPRSS2 protein in Western blot analysis **B** and the mRNA levels of indicated genes in RT-qPCR analysis **C**–**J** respectively. * *p* < 0.05, ** *p* < 0.01, *** *p* <0.001, and **** *p *< 0.0001.

**Additional file 4: Fig. S4 **Umbelliferone represses Omicron BA.2-associated pulmonary inflammation *in vivo*.The mice infected with VSVG or Omicron BA.2 pseudoviruses were sacrificed after oral administration with sterilized water, 70 mg/kg umbelliferone, and 10 mg/kg cepharanthinefor 9 days, and lungs were subsequently harvested for the examinations of histopathological changes in H&E staining **A** lymphocyte infiltration and TNF-α protein expression in IHC staining **A** TMPRSS2 protein in Western blot analysis **B** and the mRNA levels of indicated genes in RT-qPCR analysis **C**–**J** respectively. * *p* < 0.05, ** *p* < 0.01, and *** *p* <0.001. The granuloma formation is marked with a circle.

**Additional file 5: Fig. S5** Omicron variants strongly elicited the expressions of inflammatory cytokines and fibrosis markers *in vivo*.. The mice infected with wild-type SARS-CoV-2 and Omicron variantspseudo lentiviruses were sacrificed, and total RNA extracted from lung tissues were subjected to RT-qPCR analyses for the mRNA levels of inflammatory cytokines, including Tnf **A** Ifng **B** Il1b **C** Il6 **D** Il10 **E** and Tgfb1 **F** and lung fibrosis marker Acta2 **G**. * *p* < 0.05, ** *p* < 0.01, and **** *p *< 0.0001.

**Additional file 6: Fig. S6** Eriodictyol suppresses the activity of viral replication enzymes. **A** FRET-based enzymatic activity assay was performed to address the activity of RdRp **A** 3CL^pro^ **B** and PL^pro^ **C** under treatment with eriodictyol at the indicated concentrations. GS-443902, GC-376, and GRL-0617were used as the positive controls for the inhibitions of RdRp, 3CL^pro^, and PL^pro^ activities, respectively. Data were shown as mean±SEM from three independent experiments with triplicates. ** *p* < 0.01 and **** *p *< 0.0001. **D** Schematics of the working model of eriodictyol in inhibiting viral replication by antagonizing RdRp activity. The working model was created by bioRENDER.

**Additional file 7: Table S1 **The primers for determining gene expressions in this study.

## Data Availability

All of performed and analyzed results in this main article are available from the corresponding authors upon request.

## Abbreviations

*A.argyi*: *Artemisia argyi*
TCM: Traditional Chinese medicine
SARS-CoV-2: Severe acute respiratory syndrome coronavirus 2
TMPRSS2: Proteins transmembrane serine protease 2
ACE2: Angiotensin-converting enzyme 2
S protein: Spike protein
COVID-19: Coronavirus disease 2019
VOIs: Variant of interests
VOCs: Variant of concerns
WHO: World Health Organization
CoVs: Coronaviruses
FP: Fusion peptide
MERS: Middle east respiratory syndrome
13-Oxo-ODE: 13-Oxo-9E,11E-octadecadienoic acid
13-HODE: 13-Hydroxyoctadecadienoic acid
LOX: 12/15-Lipoxygenase
PPARγ: Peroxisome proliferation-activated receptor gamma
CC50: Cytotoxicity concentration 50%
SARS-CoV-2 S-Vpp: SARS-CoV-2 S protein-pseudotyped lentiviral particles
IC50: Inhibitory concentration 50%
3CL^pro^: 3-Chymotrypsin-like protease
PL^pro^: Papain-like protease
RdRp: RNA-dependent RNA polymeras
α-SMA: Alpha-smooth muscle actin
RBD: Receptor binding domain
FRET: Fluorescence resonance energy transfer
CAM: Camostat
NAP: Naphthofluorescein
GC: GC-376
GRL: GRL-0617
GS: GS-443902
TR-FRET: Time-resolved fluorescence resonance energy transfer

## References

[1] Del Rio C, Malani PN (2020). COVID-19-New insights on a rapidly changing epidemic. JAMA.

[2] Harvey WT, Carabelli AM, Jackson B, Gupta RK, Thomson EC, Harrison EM, Ludden C, Reeve R, Rambaut A, Consortium C-GU (2021). SARS-CoV-2 variants spike mutations and immune escape. Nat Rev Microbiol.

[3] Coronaviridae Study Group of the International Committee on Taxonomy of V (2020). The species severe acute respiratory syndrome-related coronavirus: classifying 2019-nCoV and naming it SARS-CoV-2. Nat Microbiol.

[4] Karim SSA, Karim QA (2021). Omicron SARS-CoV-2 variant: a new chapter in the COVID-19 pandemic. Lancet.

[5] Pulliam JRC, van Schalkwyk C, Govender N, von Gottberg A, Cohen C, Groome MJ, Dushoff J, Mlisana K, Moultrie H (2022). Increased risk of SARS-CoV-2 reinfection associated with emergence of omicron in South Africa. Science.

[6] Willett BJ, Grove J, MacLean OA, Wilkie C, De Lorenzo G, Furnon W, Cantoni D, Scott S, Logan N, Ashraf S (2022). SARS-CoV-2 omicron is an immune escape variant with an altered cell entry pathway. Nat Microbiol.

[7] Gao Y, Yan L, Huang Y, Liu F, Zhao Y, Cao L, Wang T, Sun Q, Ming Z, Zhang L (2020). Structure of the RNA-dependent RNA polymerase from COVID-19 virus. Science.

[8] Wang Q, Zhang Y, Wu L, Niu S, Song C, Zhang Z, Lu G, Qiao C, Hu Y, Yuen KY (2020). Structural and functional basis of SARS-CoV-2 entry by using human ACE2. Cell.

[9] Hoffmann M, Kleine-Weber H, Schroeder S, Krüger N, Herrler T, Erichsen S, Schiergens TS, Herrler G, Wu NH, Nitsche A (2020). SARS-CoV-2 cell entry depends on ACE2 and TMPRSS2 and Is blocked by a clinically proven protease inhibitor. Cell.

[10] Wrapp D, Wang N, Corbett KS, Goldsmith JA, Hsieh CL, Abiona O, Graham BS, McLellan JS (2020). Cryo-EM structure of the 2019-nCoV spike in the prefusion conformation. Science.

[11] Bayati A, Kumar R, Francis V, McPherson PS (2021). SARS-CoV-2 infects cells after viral entry via clathrin-mediated endocytosis. J Biol Chem.

[12] Hoffmann M, Kleine-Weber H, Pöhlmann S (2020). A multibasic cleavage site in the spike protein of SARS-CoV-2 Is essential for infection of human lung cells. Mol Cell.

[13] Shang J, Wan Y, Luo C, Ye G, Geng Q, Auerbach A, Li F (2020). Cell entry mechanisms of SARS-CoV-2. Proc Natl Acad Sci U S A.

[14] Dömling A, Gao L (2020). Chemistry and biology of SARS-CoV-2. Chem.

[15] V'Kovski P, Kratzel A, Steiner S, Stalder H, Thiel V (2021). Coronavirus biology and replication: implications for SARS-CoV-2. Nat Rev Microbiol.

[16] Luo DY, Yan ZT, Che LR, Zhu JJ, Chen B (2022). Repellency and insecticidal activity of seven *Mugwort* (*Artemisia argyi*) essential oils against the malaria vector *Anopheles*
*sinensis*. Sci Rep.

[17] Bisht D, Kumar D, Kumar D, Dua K, Chellappan DK (2021). Phytochemistry and pharmacological activity of the genus *Artemisia*. Arch Pharm Res.

[18] Nigam M, Atanassova M, Mishra AP, Pezzani R, Devkota HP, Plygun S, Salehi B, Setzer WN, Sharifi-Rad J (2019). Bioactive compounds and health benefits of *Artemisia* Species. Nat Prod Commun.

[19] Lang SJ, Schmiech M, Hafner S, Paetz C, Steinborn C, Huber R, Gaafary ME, Werner K, Schmidt CQ, Syrovets T (2019). Antitumor activity of an *Artemisia* annua herbal preparation and identification of active ingredients. Phytomedicine.

[20] Tin AS, Sundar SN, Tran KQ, Park AH, Poindexter KM, Firestone GL (2012). Antiproliferative effects of artemisinin on human breast cancer cells requires the downregulated expression of the E2F1 transcription factor and loss of E2F1-target cell cycle genes. Anticancer Drugs.

[21] Ren W, Liang P, Ma Y, Sun Q, Pu Q, Dong L, Luo G, Mazhar M, Liu J, Wang R (2021). Research progress of traditional Chinese medicine against COVID-19. Biomed Pharmacother.

[22] Zhao Z, Li Y, Zhou L, Zhou X, Xie B, Zhang W, Sun J (2021). Prevention and treatment of COVID-19 using traditional Chinese Medicine: a review. Phytomedicine.

[23] Cheng FJ, Huynh TK, Yang CS, Hu DW, Shen YC, Tu CY, Wu YC, Tang CH, Huang WC, Chen Y (2021). Hesperidin is a potential inhibitor against SARS-CoV-2 infection. Nutrients.

[24] Earles SM, Bronstein JC, Winner DL, Bull AW (1991). Metabolism of oxidized linoleic acid: characterization of 13-hydroxyoctadecadienoic acid dehydrogenase activity from rat colonic tissue. Biochim Biophys Acta.

[25] Reinaud O, Delaforge M, Boucher JL, Rocchiccioli F, Mansuy D (1989). Oxidative metabolism of linoleic acid by human leukocytes. Biochem Biophys Res Commun.

[26] Altmann R, Hausmann M, Spöttl T, Gruber M, Bull AW, Menzel K, Vogl D, Herfarth H, Schölmerich J, Falk W (2007). 13-Oxo-ODE is an endogenous ligand for PPARgamma in human colonic epithelial cells. Biochem Pharmacol.

[27] Dubuquoy L, Rousseaux C, Thuru X, Peyrin-Biroulet L, Romano O, Chavatte P, Chamaillard M, Desreumaux P (2006). PPARgamma as a new therapeutic target in inflammatory bowel diseases. Gut.

[28] Walls AC, Park YJ, Tortorici MA, Wall A, McGuire AT, Veesler D (2020). Structure, function, and antigenicity of the SARS-CoV-2 Spike glycoprotein. Cell.

[29] Guo L, Bai S, Ding S, Zhao L, Xu S, Wang X (2022). PM2.5 Exposure induces lung injury and fibrosis by regulating ferroptosis via TGF-beta signaling. Dis Markers.

[30] Báez-Santos YM, St John SE, Mesecar AD (2015). The SARS-coronavirus papain-like protease: structure, function and inhibition by designed antiviral compounds. Antiviral Res.

[31] Grum-Tokars V, Ratia K, Begaye A, Baker SC, Mesecar AD (2008). Evaluating the 3C-like protease activity of SARS-coronavirus: recommendations for standardized assays for drug discovery. Virus Res.

[32] Kirchdoerfer RN, Ward AB (2019). Structure of the SARS-CoV nsp12 polymerase bound to nsp7 and nsp8 co-factors. Nat Commun.

[33] Krause PR, Fleming TR, Longini IM, Peto R, Briand S, Heymann DL, Beral V, Snape MD, Rees H, Ropero AM (2021). SARS-CoV-2 variants and vaccines. N Engl J Med.

[34] Alam S, Sarker MMR, Afrin S, Richi FT, Zhao C, Zhou JR, Mohamed IN (2021). Traditional herbal medicines, bioactive metabolites, and plant products against COVID-19: update on clinical trials and mechanism of actions. Front Pharmacol.

[35] Wang H, Xu B, Zhang Y, Duan Y, Gao R, He H, Li X, Li J (2021). Efficacy and safety of traditional Chinese medicine in coronavirus disease 2019 (COVID-19): a systematic review and meta-analysis. Front Pharmacol.

[36] Tsai KC, Huang YC, Liaw CC, Tsai CI, Chiou CT, Lin CJ, Wei WC, Lin SJ, Tseng YH, Yeh KM (2021). A traditional Chinese medicine formula NRICM101 to target COVID-19 through multiple pathways: a bedside-to-bench study. Biomed Pharmacother.

[37] Tseng YH, Lin SJ, Hou SM, Wang CH, Cheng SP, Tseng KY, Lee MY, Lee SM, Huang YC, Lin CJ (2022). Curbing COVID-19 progression and mortality with traditional Chinese medicine among hospitalized patients with COVID-19: A propensity score-matched analysis. Pharmacol Res.

[38] Lu Y, Zhang B, Wang N, Li M, Xi N (2022). Investigation of major flavonoids from *Artemisia argyi* as a potential COVID-19 drug: molecular docking and DFT calculations. Crystals.

[39] Song X, Wen X, He J, Zhao H, Li S, Wang M (2019). Phytochemical components and biological activities of*Artemisia argyi*. Journal of Functional Foods.

[40] Cojocaru C, Cojocaru E, Turcanu AM, Zaharia DC (2022). Clinical challenges of SARS-CoV-2 variants (Review). Exp Ther Med.

[41] Socher E, Conrad M, Heger L, Paulsen F, Sticht H, Zunke F, Arnold P (2021). Mutations in the B117 SARS-CoV-2 spike protein reduce receptor-binding affinity and induce a flexible link to the fusion peptide. Biomedicines.

[42] Niu Z, Zhang Z, Gao X, Du P, Lu J, Yan B, Wang C, Zheng Y, Huang H, Sun Q (2021). N501Y mutation imparts cross-species transmission of SARS-CoV-2 to mice by enhancing receptor binding. Signal Transduct Target Ther.

[43] Vimalanathan S, Shehata M, Sadasivam K, Delbue S, Dolci M, Pariani E, D'Alessandro S, Pleschka S (2022). Broad antiviral effects of *Echinacea* purpurea against SARS-CoV-2 variants of concern and potential mechanism of action. Microorganisms.

[44] Luedemann M, Stadler D, Cheng CC, Protzer U, Knolle PA, Donakonda S (2022). Montelukast is a dual-purpose inhibitor of SARS-CoV-2 infection and virus-induced IL-6 expression identified by structure-based drug repurposing. Comput Struct Biotechnol J.

[45] Viana R, Moyo S, Amoako DG, Tegally H, Scheepers C, Althaus CL, Anyaneji UJ, Bester PA, Boni MF, Chand M (2022). Rapid epidemic expansion of the SARS-CoV-2 omicron variant in southern Africa. Nature.

[46] Mannar D, Saville JW, Zhu X, Srivastava SS, Berezuk AM, Tuttle KS, Marquez AC, Sekirov I, Subramaniam S (2022). SARS-CoV-2 omicron variant: antibody evasion and cryo-EM structure of spike protein-ACE2 complex. Science.

[47] Peacock TP, Brown JC, Zhou J, Thakur N, Newman J, Kugathasan R, Sukhova K, Kaforou M, Bailey D, Barclay WS. The SARS-CoV-2 variant, Omicron, shows rapid replication in human primary nasal epithelial cultures and efficiently uses the endosomal route of entry. bioRxiv doi: 10.1101/2021.12.31.474653

[48] Zhao H, Lu L, Peng Z, Chen LL, Meng X, Zhang C, Ip JD, Chan WM, Chu AW, Chan KH (2022). SARS-CoV-2 Omicron variant shows less efficient replication and fusion activity when compared with delta variant in TMPRSS2-expressed cells. Emerg Microbes Infect.

[49] Hu Y, Ma C, Szeto T, Hurst B, Tarbet B, Wang J (2021). Boceprevir, calpain Inhibitors II and XII, and GC-376 have broad-spectrum antiviral activity against coronaviruses. ACS Infect Dis.

[50] Cheng YW, Chao TL, Li CL, Chiu MF, Kao HC, Wang SH, Pang YH, Lin CH, Tsai YM, Lee WH (2020). Furin inhibitors block SARS-CoV-2 spike protein cleavage to suppress virus production and cytopathic effects. Cell Rep.

[51] Chen Y, Huang WC, Yang CS, Cheng FJ, Chiu YF, Chen HF, Huynh TK, Huang CF, Chen CH, Wang HC (2021). Screening strategy of TMPRSS2 inhibitors by FRET-based enzymatic activity for TMPRSS2-based cancer and COVID-19 treatment. Am J Cancer Res.

[52] Fu Z, Huang B, Tang J, Liu S, Liu M, Ye Y, Liu Z, Xiong Y, Zhu W, Cao D (2021). The complex structure of GRL0617 and SARS-CoV-2 PLpro reveals a hot spot for antiviral drug discovery. Nat Commun.

[53] Cox RM, Wolf JD, Lieber CM, Sourimant J, Lin MJ, Babusis D, DuPont V, Chan J, Barrett KT, Lye D (2021). Oral prodrug of remdesivir parent GS-441524 is efficacious against SARS-CoV-2 in ferrets. Nat Commun.

[54] Fan H, He ST, Han P, Hong B, Liu K, Li M, Wang S, Tong Y (2022). Cepharanthine: a promising old drug against SARS-CoV-2. Adv Biol.

[55] Chen Y, Yang WH, Chen HF, Huang LM, Gao JY, Lin CW, Wang YC, Yang CS, Liu YL, Hou MH (2022). Tafenoquine and its derivatives as inhibitors for the severe acute respiratory syndrome coronavirus 2. J Biol Chem.

[56] Tseng SH, Lam B, Kung YJ, Lin J, Liu L, Tsai YC, Ferrall L, Roden RBS, Wu TC, Hung CF (2021). A novel pseudovirus-based mouse model of SARS-CoV-2 infection to test COVID-19 interventions. J Biomed Sci.

