# Supplementary figures and images for: Umbelliferone and eriodictyol suppress the cellular entry of SARS-CoV-2
Source: Cell Biosci. 2023 Jun 28;13:118. doi: 10.1186/s13578-023-01070-y (PMC10304356; doi:10.1186/s13578-023-01070-y)

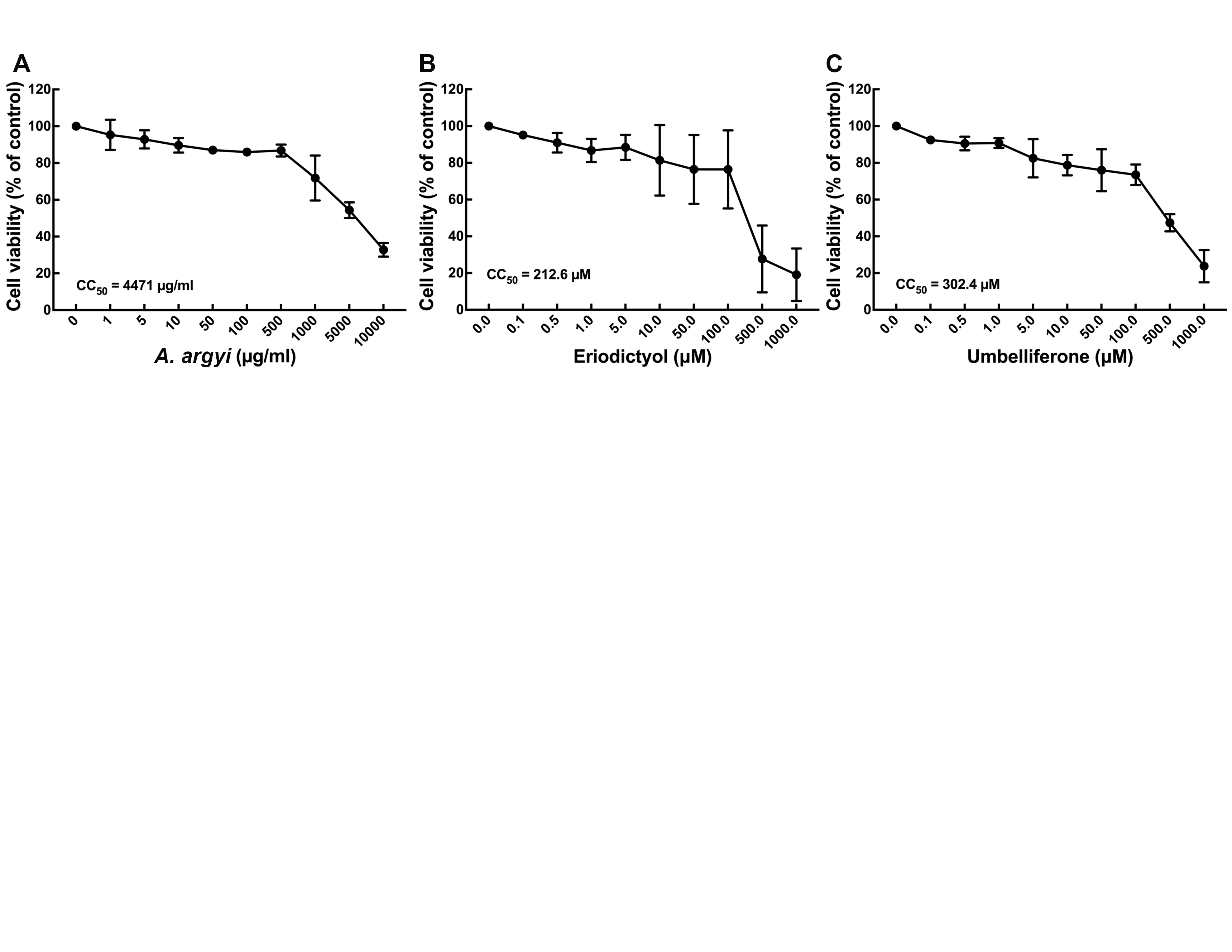

Supplement: Supplementary file 1 — Additional file 1: Fig. S1 The cytotoxicity of A. argyi, eriodictyol, and umbelliferone is illustrated in lung epithelial cells. Beas 2B cells were treated with A. argyi A eriodictyol B, and umbelliferone C at the indicated concentrations for 2 days. Then the cell viability was detected using MTT assays. The cytotoxicity concentration 50%value was calculated and displayed. Data was shown as mean±SEM from three independent experiments with triplicates. [file 13578_2023_1070_MOESM1_ESM.tif]

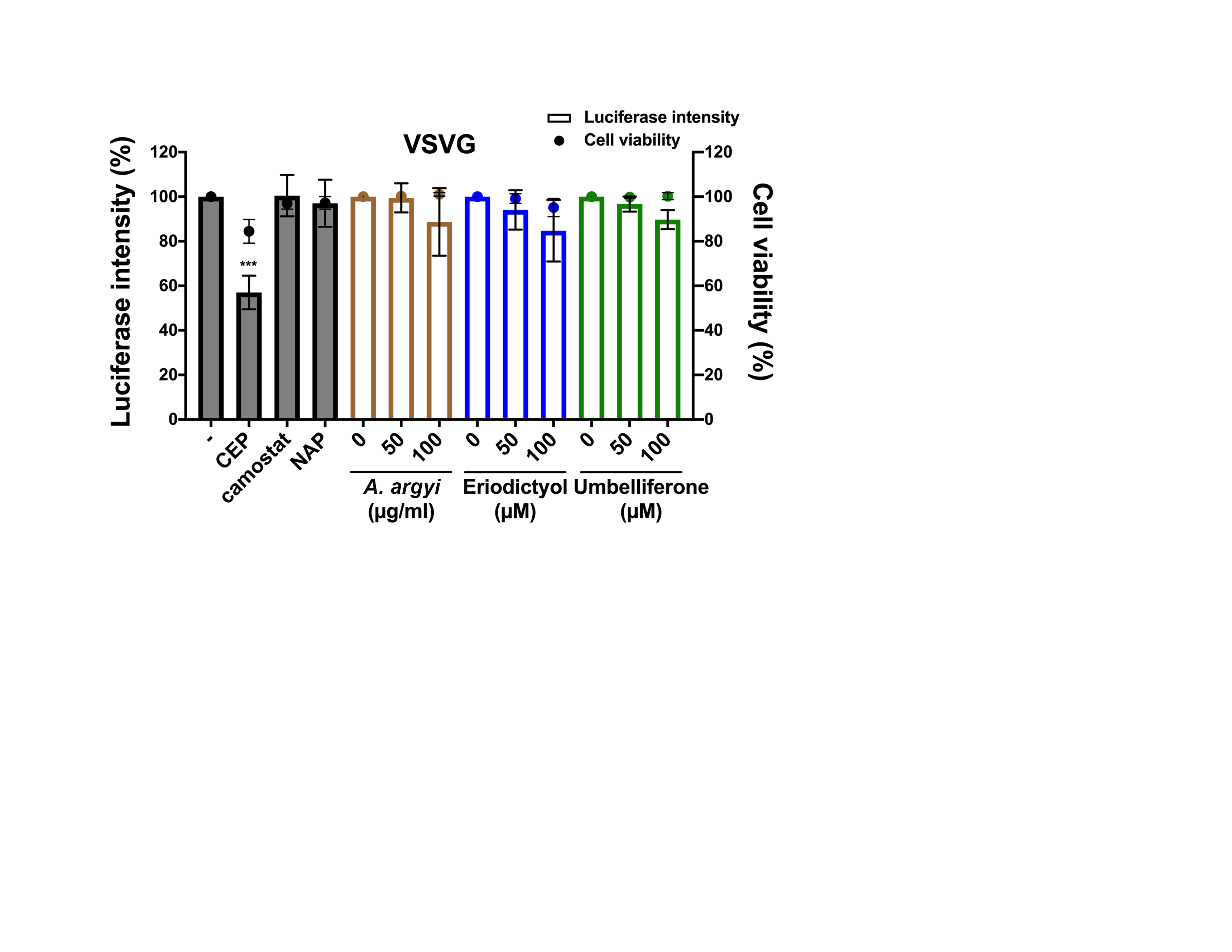

Supplement: Supplementary file 2 — Additional file 2: Fig. S2 A. argyi, eriodictyol, and umbelliferone did not affect the cellular entry of VSVG-pseudotyped lentivirus.HEK-293T cells expressing ACE2 were infected with VSVG-pseudotyped lentivirus encoding luciferase after treatments with A. argyi, eriodictyol, or umbelliferone at the indicated concentrations for 2 days, and the luciferase activities were subsequently measured to determine the infection rate with the pseudoviruses. Additionally, the cell viability also was examined in CCK-8 assays. Camostat, naphthofluorescein, and cepharanthinewere used as the positive controls for the inhibitions of TMPRSS2 and furin activities and the binding between S protein and ACE2, respectively. Data were shown as mean±SEM from three independent experiments with triplicates. *** p<0.001. [file 13578_2023_1070_MOESM2_ESM.tif]

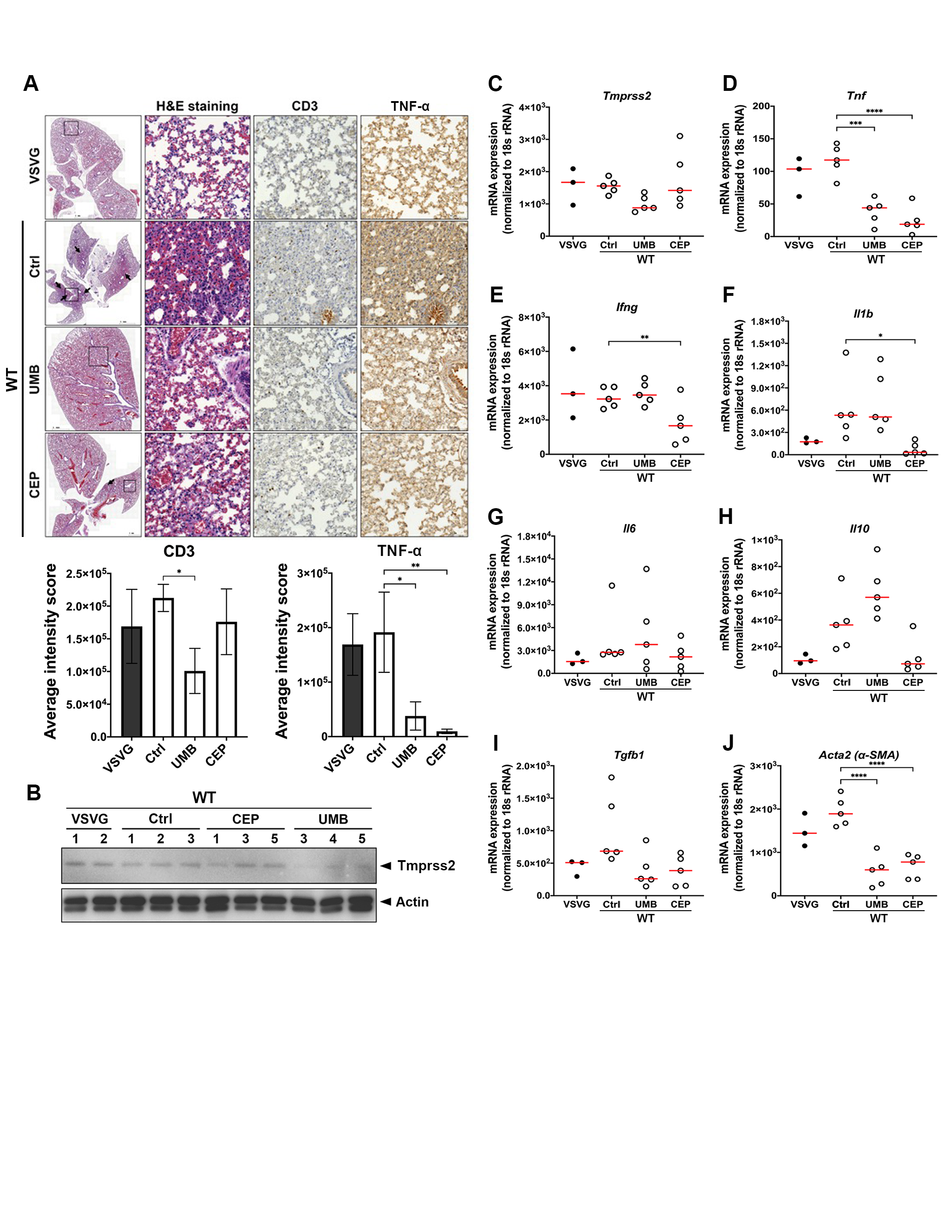

Supplement: Supplementary file 3 — Additional file 3: Fig. S3 Umbelliferone represses the wild-type SARS-CoV-2-associated pulmonary inflammation in vivo.The mice infected with VSVG or wild-type SARS-CoV-2 pseudoviruses were sacrificed after oral administration with sterilized water, 70 mg/kg umbelliferone, and 10 mg/kg cepharanthinefor 9 days, and lungs were subsequently harvested for the examinations of histopathological changes in H&E staining A lymphocyte infiltration and TNF-α protein expression in IHC staining A TMPRSS2 protein in Western blot analysis B and the mRNA levels of indicated genes in RT-qPCR analysis C–J respectively. * p < 0.05, ** p < 0.01, *** p <0.001, and **** p < 0.0001. [file 13578_2023_1070_MOESM3_ESM.tif]

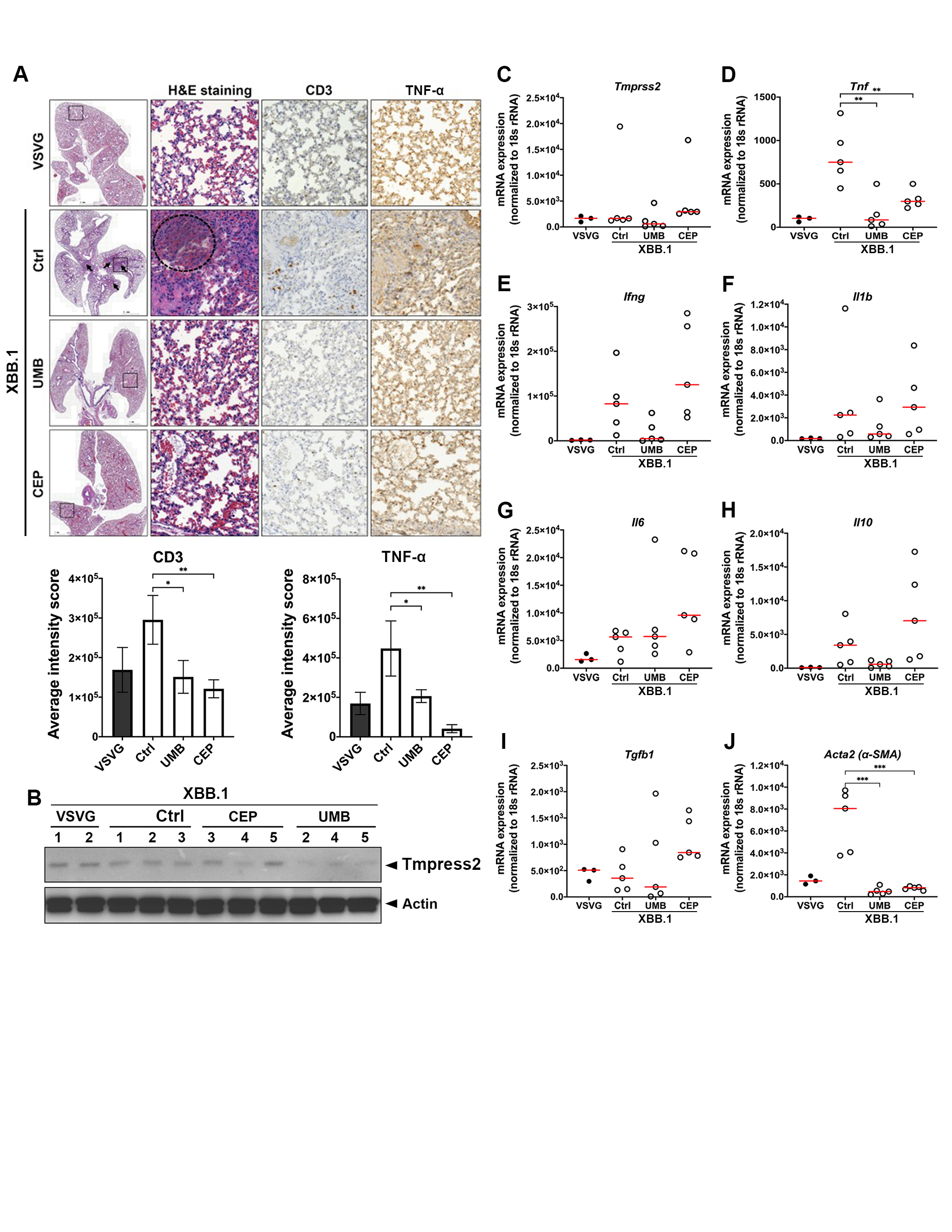

Supplement: Supplementary file 4 — Additional file 4: Fig. S4 Umbelliferone represses Omicron BA.2-associated pulmonary inflammation in vivo.The mice infected with VSVG or Omicron BA.2 pseudoviruses were sacrificed after oral administration with sterilized water, 70 mg/kg umbelliferone, and 10 mg/kg cepharanthinefor 9 days, and lungs were subsequently harvested for the examinations of histopathological changes in H&E staining A lymphocyte infiltration and TNF-α protein expression in IHC staining A TMPRSS2 protein in Western blot analysis B and the mRNA levels of indicated genes in RT-qPCR analysis C–J respectively. * p < 0.05, ** p < 0.01, and *** p <0.001. The granuloma formation is marked with a circle. [file 13578_2023_1070_MOESM4_ESM.tif]

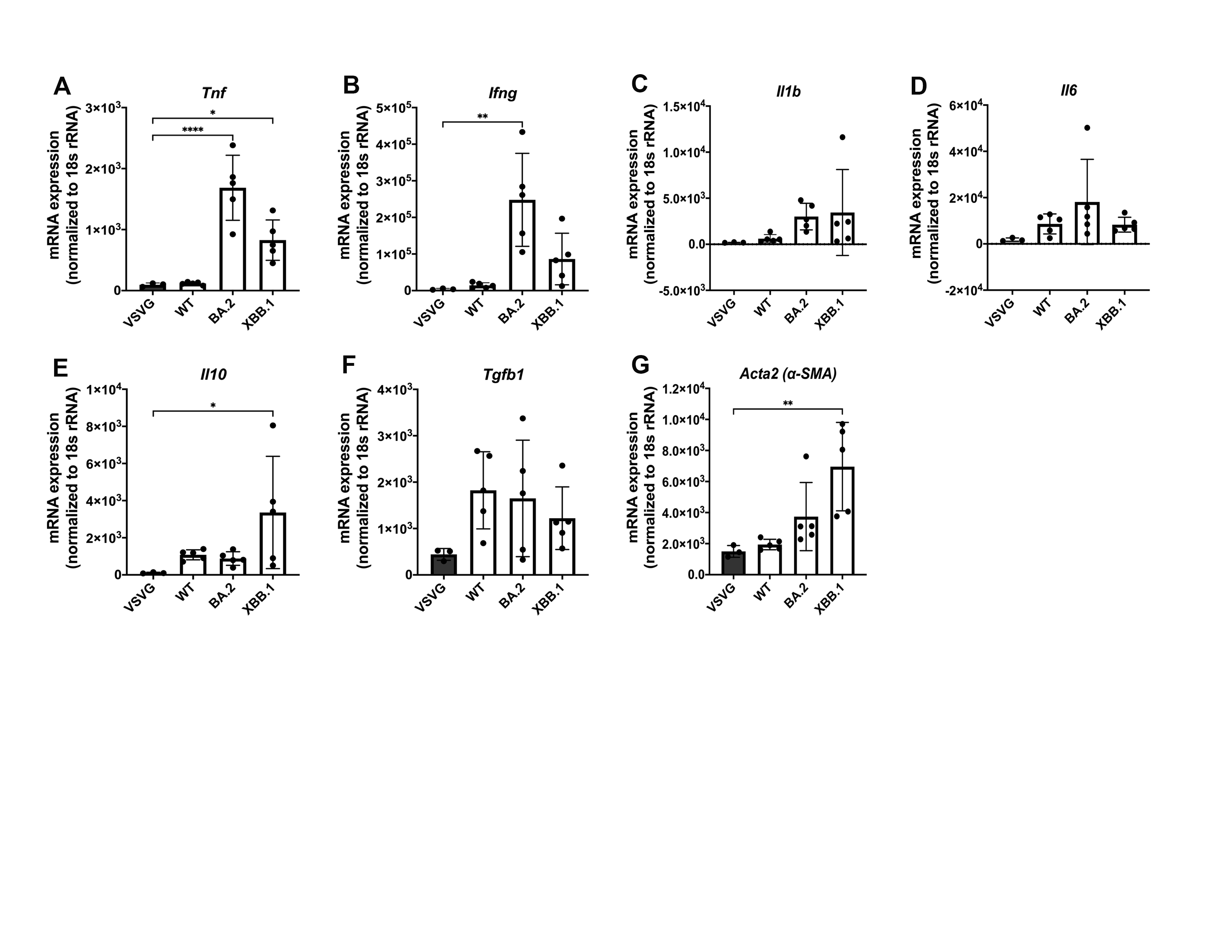

Supplement: Supplementary file 5 — Additional file 5: Fig. S5 Omicron variants strongly elicited the expressions of inflammatory cytokines and fibrosis markers in vivo.. The mice infected with wild-type SARS-CoV-2 and Omicron variantspseudo lentiviruses were sacrificed, and total RNA extracted from lung tissues were subjected to RT-qPCR analyses for the mRNA levels of inflammatory cytokines, including Tnf A Ifng B Il1b C Il6 D Il10 E and Tgfb1 F and lung fibrosis marker Acta2 G. * p < 0.05, ** p < 0.01, and **** p < 0.0001. [file 13578_2023_1070_MOESM5_ESM.tif]

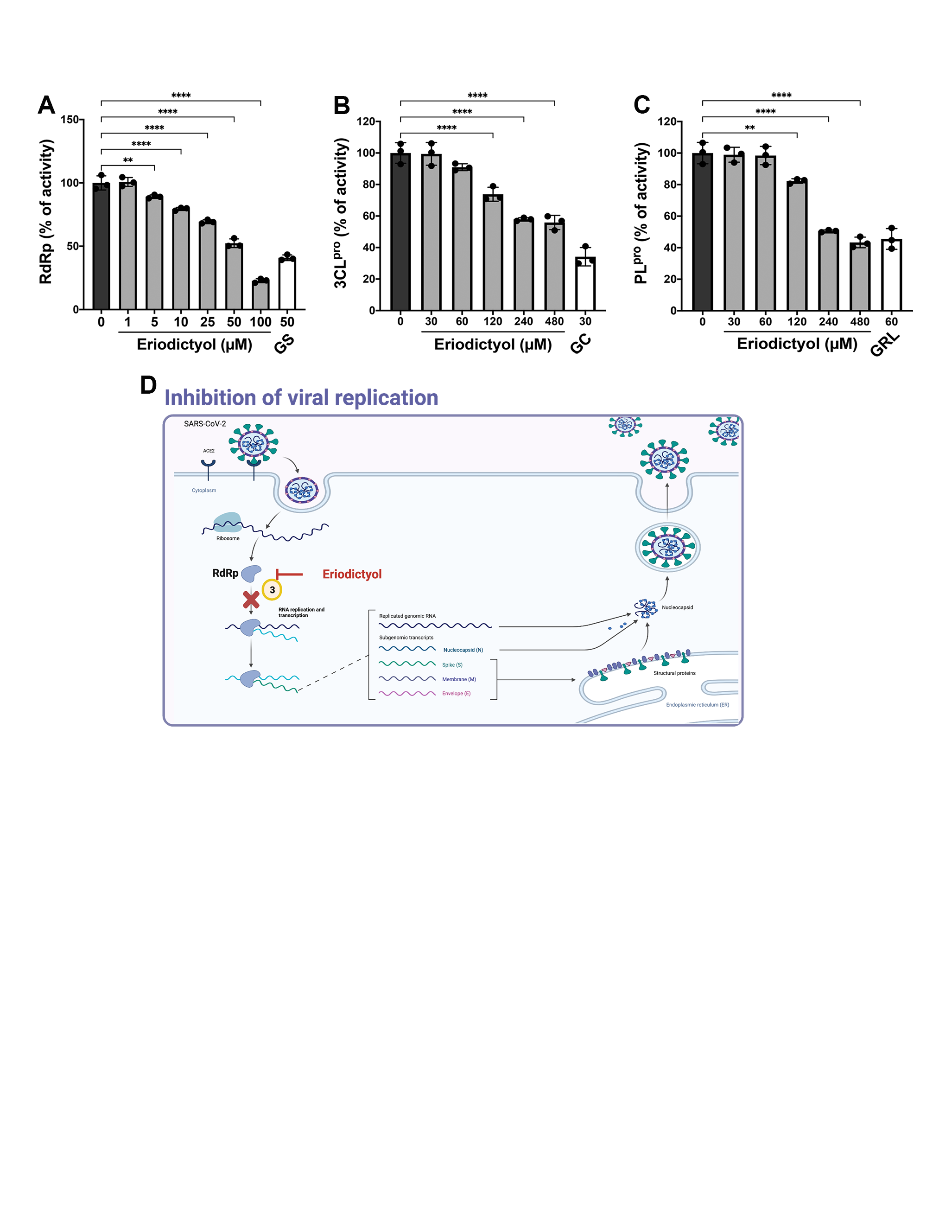

Supplement: Supplementary file 6 — Additional file 6: Fig. S6 Eriodictyol suppresses the activity of viral replication enzymes. A FRET-based enzymatic activity assay was performed to address the activity of RdRp A 3CLpro B and PLpro C under treatment with eriodictyol at the indicated concentrations. GS-443902, GC-376, and GRL-0617were used as the positive controls for the inhibitions of RdRp, 3CLpro, and PLpro activities, respectively. Data were shown as mean±SEM from three independent experiments with triplicates. ** p < 0.01 and **** p < 0.0001. D Schematics of the working model of eriodictyol in inhibiting viral replication by antagonizing RdRp activity. The working model was created by bioRENDER. [file 13578_2023_1070_MOESM6_ESM.tif]
