# Supplementary material for: Umbelliferone and eriodictyol suppress the cellular entry of SARS-CoV-2
Source: Cell Biosci. 2023 Jun 28;13:118. doi: 10.1186/s13578-023-01070-y (PMC10304356; doi:10.1186/s13578-023-01070-y)
Supplement: Supplementary file 7 — Additional file 7: Table S1 The primers for determining gene expressions in this study. [file 13578_2023_1070_MOESM7_ESM.docx]

**Table S1 The primers for determining gene expressions in this study.**

| **Gene** | **Species** | **Forward sequence (5’ – 3’)** | **Reverse sequence (5’ – 3’)** |
| --- | --- | --- | --- |
| *ACE2* | Human | TCCATTGGTCTTCTGCCATCCG | AGACCATCCACCTCCACTTCTC |
| *TMPRSS2* | Human | CCTCTAACTGGTGTGATGGCGT | TGCCAGGACTTCCTCTGAGATG |
| *Tmprss2* | Mouse | AAGTCCTCAGGAGCACTGTGCA | CAGAACCTCCAAAGCAAGACAGC |
| *Tnf* | Mouse | GGTGCCTATGTCTCAGCCTCTT | GCCATAGAACTGATGAGAGGGAG |
| *Ifng* | Mouse | CAGCAACAGCAAGGCGAAAAAGG | TTTCCGCTTCCTGAGGCTGGAT |
| *Il1b* | Mouse | TGGACCTTCCAGGATGAGGACA | GTTCATCTCGGAGCCTGTAGTG |
| *Il6* | Mouse | TACCACTTCACAAGTCGGAGGC | CTGCAAGTGCATCATCGTTGTTC |
| *Il10* | Mouse | CGGGAAGACAATAACTGCACCC | CGGTTAGCAGTATGTTGTCCAGC |
| *Tgfb1* | Mouse | TGATACGCCTGAGTGGCTGTCT | CACAAGAGCAGTGAGCGCTGAA |
| *Acta2* | Mouse | TGCTGACAGAGGCACCACTGAA | CAGTTGTACGTCCAGAGGCATAG |
